# Biological Activities of Cyclic and Acyclic B-Type Laxaphycins in SH-SY5Y Human Neuroblastoma Cells

**DOI:** 10.3390/md18070364

**Published:** 2020-07-15

**Authors:** Rebeca Alvariño, Eva Alonso, Louis Bornancin, Isabelle Bonnard, Nicolas Inguimbert, Bernard Banaigs, Luis M. Botana

**Affiliations:** 1 Departamento de Farmacología, Facultad de Veterinaria, Universidad de Santiago de Compostela, 27003 Lugo, Spain; rebeca.alvarino@usc.es (R.A.); luis.botana@usc.es (L.M.B.); 2 Fundación Instituto de Investigación Sanitario Santiago de Compostela (FIDIS), Hospital Universitario Lucus Augusti, 27003 Lugo, Spain; 3 PSL Research University: EPHE-UPVD-CNRS, USR 3278 CRIOBE, Université de Perpignan, 52 Avenue Paul Alduy, 66860 Perpignan, France; lbornan@dtu.dk (L.B.); isabelle.bonnard@univ-perp.fr (I.B.); nicolas.inguimbert@univ-perp.fr (N.I.); banaigs@univ-perp.fr (B.B.); 4 Laboratoire d’Excellence “CORAIL”, Université de Perpignan Via Domitia, 58 Avenue Paul Alduy, 66860 Perpignan, France

**Keywords:** biotransformation, laxaphycin, autophagy, apoptosis, cyanobacteria

## Abstract

Laxaphycins are a family of non-ribosomal lipopeptides that have been isolated from several cyanobacteria. Some of these compounds have presented cytotoxic activities, but their mechanism of action is poorly understood. In this work, the already described laxaphycins B and B3, and acyclolaxaphycins B and B3 were isolated from the marine cyanobacteria *Anabaena torulosa*. Moreover, two new acyclic compounds, [des-(Ala^4^-Hle^5^)] acyclolaxaphycins B and B3, were purified from the herviborous gastropod *Stylocheilus striatus*, with this being the first description of biotransformed laxaphycins. The structure of these new compounds was elucidated, together with the absolute configuration of acyclolaxaphycins B and B3. The bioactivities of the six peptides were determined in SH-SY5Y human neuroblastoma cells. Laxaphycins B and B3 were cytotoxic (IC_50_: 1.8 and 0.8 µM, respectively) through the induction of apoptosis. In comparison, acyclic laxaphycins did not show cytotoxicity but affected mitochondrial functioning, so their effect on autophagy-related protein expression was analyzed, finding that acyclic peptides affected this process by increasing AMPK phosphorylation and inhibiting mTOR. This work confirms the pro-apoptotic properties of cyclic laxaphycins B and is the first report indicating the effects on autophagy of their acyclic analogs. Moreover, gastropod-derived compounds presented ring opening and amino-acids deletion, a biotransformation that had not been previously described.

## 1. Introduction

The phylum Cyanobacteria includes photosynthetic prokaryotes from terrestrial, freshwater and marine ecosystems. These Gram-negative organisms, also called blue-green algae, appeared on Earth over 3.5 billion years ago and can live as colonial or unicellular forms in almost all habitats (deserts, ice shelves, as endosymbionts, etc.). This high degree of adaptation is related to their ability to produce a wide range of secondary metabolites [1,2]. A significant part of these compounds are complex cyclic peptides, depsipeptides or lipopeptides that contain unusual amino acids and multiple N-methylations [3]. Many of these molecules are produced through non-ribosomal peptide synthase (NRPS) and/or polyketide synthase (PKS) enzymatic systems. The enzymes are organized in modules, each one carrying out the addition of a subpart of the final molecule and generating a great diversity of compounds [4,5]. As a result, non-ribosomal peptides exhibit a broad spectrum of biological activities, including, anticancer, antifungal and antimicrobial properties [6]. Furthermore, cyclic peptides are less flexible than their linear counterparts, and contain non-proteinogenic amino acids, two characteristics that give them greater selectivity and better resistance to hydrolysis by exo- and endopeptidases.

Laxaphycins are a large family of lipopeptides synthetized through a hybrid PKS/NRPS pathway by different species of cyanobacteria [7]. These compounds have been obtained from specimens of *Hormothamnion enteromorphoides*, *Anabaena torulosa*, *Lyngbya confervoides* and *Anabaena laxa* collected worldwide. The fact that different species could produce similar metabolites suggests horizontal gene transfer or an ancient common parent between them. Therefore, these peptides have been selected by evolution in both freshwater and oceanic cyanobacteria and may confer to them an ecological advantage. Laxaphycins are divided in two sub-families: laxaphycin A-type, undecapeptides with a segregation of hydrophobic and hydrophilic residues; and laxaphycin B-type, dodecapeptides in which hydrophobic and hydrophilic residues are alternated [8]. Laxaphycin B-type members such as laxaphycins B, B2 and B3 have presented antifungal, antimicrobial and cytotoxic activities [9,10,11,12]. Laxaphycin A-type compounds have shown weak cytotoxicity, with the exception of the compound hormothamnin A [13]. Moreover, we have recently reported evidence that points to an activation of the autophagic flux by laxaphycin A peptides [14].

Autophagy is a regulated process that leads to the clearance of misfolded or damaged proteins and dysfunctional organelles. This cellular machinery is activated by a variety of signals such as nutrient starvation, oxidative stress and energy depletion. Cells can degrade damaged components and restore substrates for energy metabolism through this pathway [15]. At basal levels, autophagy maintains cellular homeostasis and is an important mechanism in cell growth and development. This process plays an important role in many pathologies such as cancer, diabetes and neurodegenerative diseases [16]. Therefore, much effort has been made in the search for new compounds capable of targeting the autophagic flux [17,18].

When the autophagic flux is initiated, the mammalian target of rapamycin (mTOR), considered the master cell growth regulator, is inhibited. This inhibition leads to the activation of the Unc-51-like kinase 1 (ULK1) complex, which in turn stimulates the Beclin1-VPS34 complex. The components of this complex are phosphorylated and trigger the elongation of the phagophore. Two systems control this process: the ATG5-ATG12 and the microtubule-associated light chain 3 (LC3). During this step, LC3I is converted to LC3II, the lipidated form, considered the signature of the autophagic membranes [19]. Finally, the autophagosome undergoes maturation and fuses with the lysosome, leading to the formation of the autolysosome, with an internal acidic and hydrolytic environment that degrades the damaged cellular components [20,21].

Adenosine monophosphate-activated protein kinase (AMPK) is an important energy sensor in cells and plays a key role in the activation of autophagy due to the significance of this process in the generation of metabolic intermediates to maintain ATP levels. AMPK is activated when energy levels decrease and can trigger autophagic flux either by phosphorylating the ULK1 complex or through the regulation of mTOR activity [22].

The human neuroblastoma cell line SH-SY5Y is a useful model for assessing neurotoxic and neuroprotective compounds, since these cells express intact genes implied in reactive oxygen species (ROS) metabolism, or calcium and mitochondrial signaling [23].

**Figure 1:**
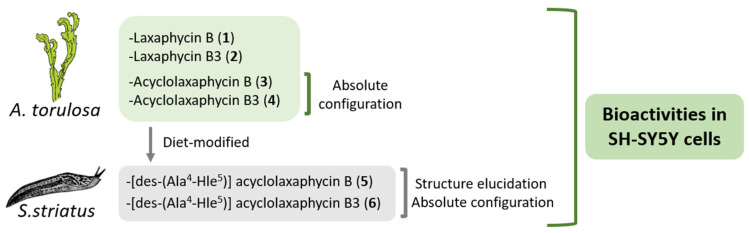
Scheme of laxaphycin B sources and compound analysis

In this study, the biological activities of six B-type laxaphycins were analyzed in SH-SY5Y cells (Figure 1). The already known laxaphycins B (**1**) and B3 (**2**), and acyclolaxaphycins B (**3**) and B3 (**4**) were obtained from a marine specimen of *A. torulosa*, whereas two new acyclic compounds, [des-(Ala^4^-Hle^5^)] acyclolaxaphycin B (**5**) and [des-(Ala^4^-Hle^5^)] acyclolaxaphycin B3 (**6**), were isolated from the herbivorous gastropod *Stylocheilus striatus*. The absolute configurations of **3** and **4**, as well as the complete structural elucidation of the new diet-derived peptides **5** and **6** are also provided.

## 2. Results

### 2.1. Structure Elucidation of Peptides ***3***, ***4***, ***5*** and ***6***

**Figure 2:**
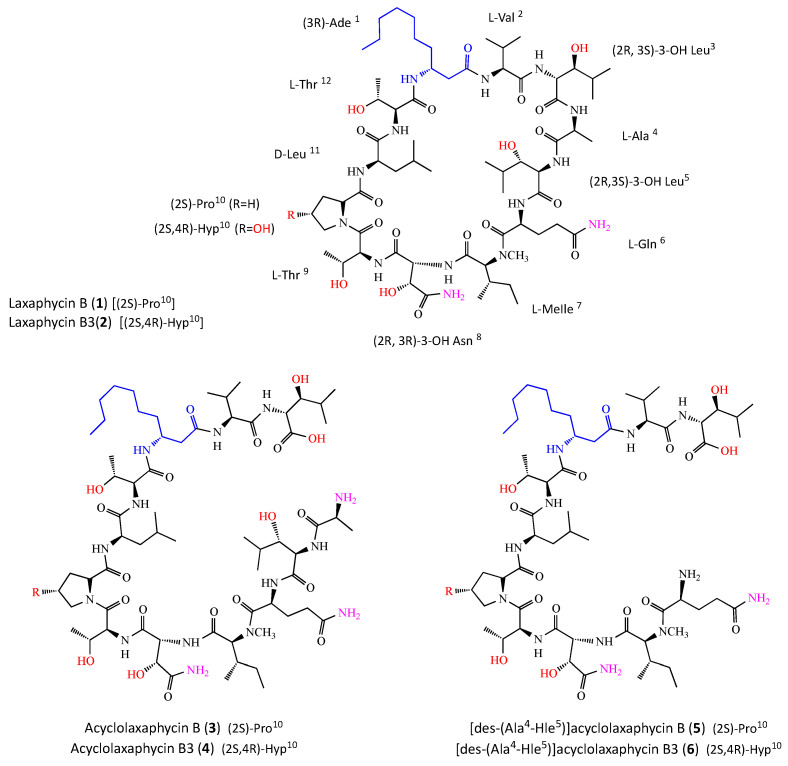
Chemical structures of laxaphycin B-type compounds. As for (**1**) and (**2**), R=H for (**3**) and (**5**), and R=OH for (**4**) and (**6**).

*S. striatus* was collected on *A*. cf *torulosa* in the lagoon of Moorea, French Polynesia, sealed underwater in a bag, freeze-dried and extracted. The crude extract was fractionated using flash chromatography and the resulting fraction containing new peptides was subjected to HPLC purification to yield compounds **5** (2.5 mg) and **6** (5 mg) as a white, amorphous powder. Compounds **5** and **6** responded positively to a ninhydrin test, suggesting a non-blocked N-terminus. The already described laxaphycins B (**1**) and B3 (**2**) [9], and acyclolaxaphycins B (**3**) and B3 (**4**) were repurified from *A. torulosa* as described in [7] (Figure 2).

Using the positive high-resolution electrospray ionization mass spectrometry (HRESIMS) spectra, the molecular formula was determined to be C_56_H_100_N_12_O_17_ (*m/z* 1213.7509) [M + H]^+^) for compound **5** and C_56_H_100_N_12_O_18_ (*m/z* 1229.7972) [M + H]^+^) for compound **6**.

All the NMR experiments were conducted in DMSO-*d6*. The signal distribution pattern observed in the ^1^H-NMR spectrum of **5** and **6** was characteristic of lipopeptides, displaying amide NH signals (δ_H_7.40–7.90), CαH signals (δ_H_3.5–4.7), aliphatic CH2 (δ_H_1.1–1.3) and CH_3_ signals (δ_H_0.7–0.9).

• [des-(Ala^4^-Hle^5^)] acyclolaxaphycin B (**5**)

In the NH proton region, seven doublets and two singlets were observed. The values of chemical shifts (Table 1) were reported using 2D-NMR spectra including correlation spectroscopy (COSY), total correlation spectroscopy (TOCSY),rotating frame nuclear magnetic resonance spectroscopy (ROESY), heteronuclear single quantum correlation (HSQC), HSQC-TOCSY, and heteronuclear multiple bond correlation (HMBC) (Figures S1–S8). Analysis of TOCSY correlations (Figure S4) revealed the presence of 10 amino acid residues: N-methylisoleucine (N-MeIle), 3-hydroxyasparagine (Has), two threonines (Thr), proline (Pro), leucine (Leu), *β*-aminodecanoic acid (*β*-Ade), valine (Val) and 3-hydroxyleucine (Hle). In comparison with laxaphycin B, analyses of TOCSY and HSQC (Figure S6) spectra revealed the absence of typical correlations within alanine and within one of the two hydroxyleucines, suggesting the lack of these two residues in **5**. 

The remaining non-identified spin system was identified as a glutamine residue with the presence of two carbonyl signals at 172.85 and 174.24 ppm, and by HMBC (Figure S8) correlation from Hα (δ_H_4.50) to Cβ (δ_C_29.95), from Hβ (δ_H_1.88) to Cα (δ_C_53.01) and Cγ (δ_C_28.76) and from Hγ (δ_H_2.20) to Cβ (δ_C_29.95) and Cδ (δ_C_174.24). However, the poor resolutions of glutamine correlation signals in 2D-NMR (HMBC, HSQC and ROESY) did not allow us to assign the two NH_2_ signals. This might be explained by the presence of multiple conformations in solution due to an increase in the flexibility of the N-terminal residue. The HMBC spectrum provided information on sequence-specific assignments. Indeed, the cross-peaks between carbonyl carbons (residue i) and NH, NCH_3_ protons or Hα (residue i + 1) suggested the presence of two partial sequences including Gln-N-MeIle-Has-Thr (fragment 1) and Pro-Leu-Thr-β-Ade-Val-Hle (fragment 2). Analysis of the ROESY spectra (Figure S5) revealed a correlation between Hα (δ_H_4.57) of Thr^4^ and Hδ (δ_H_3.64/3.76) of Pro^5^, assembling fragments 1 and 2 and establishing the complete sequence as Gln-N-MeIle-Has-Thr-Pro-Leu-Thr-β-Ade-Val-Hle. Even if it does not constitute evidence, the lack of HMBC or ROESY correlations between Gln and Hle, observable in the case of laxaphycins B (**1)** and B3 (**2**), suggested that the peptide is linear. Positive arguments are provided by the molecular formula determined to be C_56_H_100_N_12_O_17_ by HRMS and by a positive response to a ninhydrin test suggesting a non-blocked N-terminus.

• [des-(Ala^4^-Hle^5^)] acyclolaxaphycin B3 (**6**)

ESIMS-MS fragmentation of compound **6** led us to a y6-y8 ion series shifted to a higher mass by 16 amu in comparison to that of **5**, suggesting that the variable residue could be Pro. Compound **6** showed remarkable NMR spectral similarities (Figures S9–S16) to **5**, being the most significant differences being the presence of an additional hydroxyl group on proline (Hγ at 4.29 ppm vs. two Hγ at 1.81 and 1.89 ppm for [des-(Ala^4^-Hle^5^)] acyclolaxaphycin B (**5**); Cγ at 68.41 ppm vs. 24.07 ppm for **5**; Cβ and Cδ were also deblinded by the presence of the hydroxyl function (Δδ 8.8 and 8.4 ppm, respectively)). The NMR spectral analysis (Table 1) established the complete sequence as Gln-N-MeIle-Has-Thr-Hyp-Leu-Thr-β-Ade-Val-Hle.

**Figure 3:**
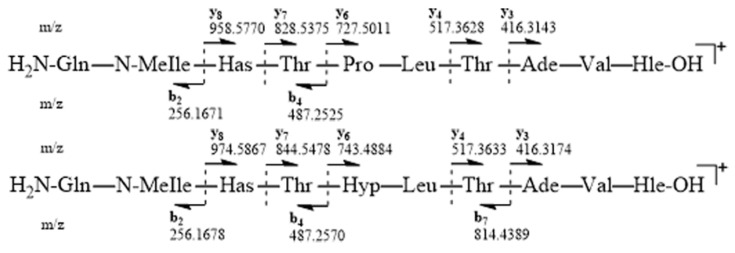
Electrospray ionization mass spectrometry (ESIMS/MS) fragmentation of [des-(Ala4-Hle5)] acyclolaxaphycins B (**5**) and B3 (**6**)

ESIMS-MS fragmentations for **5** and **6** were consistent with the proposed amino acid sequences and the presence of y ions at *m/z* 416.3143 (y3), 517.3628 (y4), 727.5011 (y6), 828.5375 (y7) and 958.5770 (y8), and b ions at *m/z* 487.2525 (b4) and 256.1671 (b2) for compound **5**, as well as y ions at *m/z* 416.3174 (y3), 517.3633 (y4), 743.4884 (y6), 844.5478 (y7) and 974.5867 (y8), and b ions at *m/z* 814.4389 (b7), 487.2570 (b4) and 256.1678 (b2) for compound **6** (Figure 3).

### 2.2. Absolute Configuration of Peptides ***3***, ***4***, ***5*** and ***6***

The absolute configuration of each amino acid residue in compounds **3**–**6** was established using the advanced Marfey’s method after hydrolysis [24,25]. The LC-MS comparison between the Marfey’s derivatives of the acid hydrolysate of laxaphycin B and those of acyclolaxaphycin B (**3**) established the 2*S* configuration of Val, Ala, Gln, Pro, N-MeIle, the 2*R* configuration of Leu, as well as the 3*R* configuration of Ade. The Marfey’s analysis of the four stereoisomers of standard threonine revealed the (*2S,3R*) configuration of both threonines present in acyclolaxaphycin B (**3**). The Marfey’s method also revealed the 2*R* configuration of the two 3-hydroxyleucines. The absolute configuration of Cβ of both 3-hydroxyleucines (2*R*,3*S*) was established through NOESY correlations between the Hγ and the NH observed.

As previously described [9], the elution order of the 3-hydroxyasparagine (HAsp), which results from the acid hydrolysis of Has, is another exception of Marfey’s rule. Indeed, the D-FDLA-(2*R*)-HAsp derivative elutes after the L-FDLA-(2*R*)-HAsp derivative. Thus, we established that the Cα configuration of the Has residue was 2*R*. The configuration of the Cβ of Has was established to be 3*R* by a comparison with laxaphycin B Marfey’s derivatives. Therefore, the complete structure of acyclolaxaphycin B (**3**) was established as (*2S)*- Ala-(2*R*,3*S*)- Hle-(*2S)*- Gln-(2*S*)-*N*- MeIle-(2*R*,3*R*)-Has-(*2S,3R)*-Thr-(*2S)*-Pro-(*2R)*-Leu-(*2S,3R*)-Thr-(3*R*)-Ade-(*2S)*-Val-(2*R*,3*S*)-Hle.

Regarding acyclolaxaphycin B3 (**4**), the configuration of Val, Ala, Gln, N-MeIle, Leu, Ade, Has, Thr (x2) and Hle (x2) were found to be the same as for acyclophycin B (**3**). The absolute configuration of the Cα of the Hyp residue appeared to be (2*S*) and a comparison with laxaphycin B3 derivative enabled the Cγ configuration to be assigned to *4R,* establishing the complete structure as (*2S)*-Ala-(2*R*,3*S*)-Hle-(*2S*)-Gln-(*2S*)-*N*-MeIle-(2*R*,3*R*)-Has-(*2S,3R)*-Thr-(*2S,4R)*-Hyp-(*2R)*-Leu-(*2S,3R)*-Thr-(3*R*)-Ade-(*2S*)-Val-(2*R*,3*S*)-Hle.

The chromatographic comparison between the Marfey’s derivatives of the acid hydrolysate of [des-(Ala^4^-Hle^5^)] acyclolaxaphycin B (**5**) and those of acyclophycin B (**3**) established the 2*S* configuration of Val, Gln, Pro, N-MeIle, the 2*R* configuration of Leu, as well as the 3*R* configuration of Ade, and the (2*R*,3*R*) configuration of Has, *(2S,3R)* of Thr (x2), and (2*R*,3*S*) of Hle. The complete structure of **5** was defined as (2*S*)-Gln-(2*S*)-*N*-MeIle-(2*R*,3*R*)-Has-(*2S,3R*)-Thr-(*2S)*-Pro-(*2R)*-Leu-(*2S,3R*)-Thr-(3*R*)-Ade-(2*S*)-Val-(2*R*,3*S*)-Hle.

With regard to [des-(Ala^4^-Hle^5^)] acyclolaxaphycin B3 (**6**), the configuration of Val, Gln, N-MeIle, Leu, Ade, Has, Thr (x2) and Hle (x2) were found to be the same as for **5**. The absolute configuration of the Cα of the Hyp residue appeared to be (2*S*) and a comparison with laxaphycin B3 derivative enabled the Cγ configuration to be assigned to *4R,* establishing the complete structure as (*2S)*-Gln-(*2S)*-*N*-MeIle-(2*R*,3*R*)-Has-(*2S,3R)*-Thr-(*2S,4R)*-Hyp-(*2R)*-Leu-(*2S)*-Thr-(3*R*)-Ade-(*2S)*-Val-(*2R,3S*)-Hle.

### 2.3. Effects of B-Type Laxaphycins on Cell Viability and Mitochondrial Function

In order to make an initial evaluation of compounds activity, their effect on cell viability, metabolic activity and mitochondrial function were tested. Cell viability was assessed by monitoring lactate dehydrogenase (LDH) levels in cells supernatant [26], cell metabolic activity was analyzed with MTT (3-(4, 5-dimethyl thiazol-2-yl)-2, 5-diphenyl tetrazolium bromide) [27], and tetramethylrhodamine methyl ester (TMRM) dye was used to determine the mitochondrial membrane potential (∆Ψ_m_). The effect of laxaphycins over reactive oxygen species (ROS) and ATP levels was also monitored.

**Figure 4:**
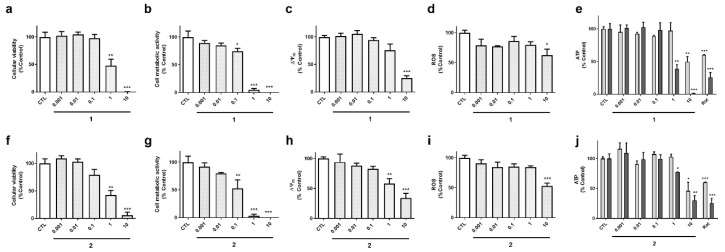
Effect of cyclic laxaphycins B on cell viability and mitochondrial function. SH-SY5Y cells were treated with compounds **1** (**a**–**e**) and **2** (**f**–**j**) for 24 h. Then, cell viability was determined with LDH test, cell metabolic activity with MTT assay, ∆Ψ_m_ with tetramethylrhodamine methyl ester (TMRM) dye, reactive oxygen species (ROS) levels with carboxy-H_2_DCFDA and ATP levels with a commercial kit. ATP levels were assessed both at 6 h (light bars) and 24 h (dark bars). Concentration expressed in µM. Mean ± SEM of three experiments performed by triplicate. Values are presented as percentage of control cells. * *p* < 0.05, ** *p* < 0.01, *** *p* < 0.001

Cyclic compounds (**1** and **2**) turned out to be cytotoxic to neuroblastoma cells (Figure 4). Their half maximal inhibitory concentrations (IC_50_) were calculated for LDH and MTT assays. In LDH test, compound **2** presented an IC_50_ value of 0.8 µM, 95% confidence interval (CI): 0.24–3.0 µM, R^2^: 0.90, being was slightly more cytotoxic than compound **1** (IC_50_=1.8 µM, 95% CI: 0.65–5.1, R^2^: 0.93) (Figure 4a,f). The same was observed in MTT assay, compound **2** was the most toxic (IC_50_ =0.15 µM, 95 % CI: 0.06–0.37 µM, R^2^: 0.91), whereas compound **1** presented an IC_50_ of 0.3 µM (CI: 0.14–0.66, R^2^: 0.94) (Figure 4b,g). As expected, cyclic laxaphycins depolarized the mitochondria and reduced ROS and ATP levels at toxic concentrations (Figure 4c–e,h–j).

**Figure 5:**
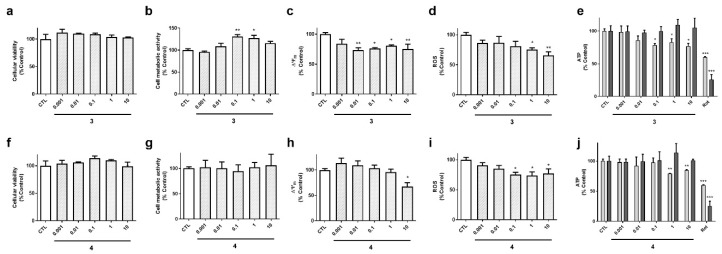
Effect of acyclic compounds isolated from *A. torulosa* on SH-SY5Y cells. Compounds **3** (**a**–**e**) and **4** (**f**–**j**) were added to neuroblastoma cells for 24 h and cellular viability, metabolic activity, ∆Ψ_m_, ROS release and ATP levels were determined. The levels of ATP were assessed at 6 and 24 h (light and dark bars, respectively). Concentration expressed in µM. Mean ± SEM of three experiments performed by triplicate. Values are presented as percentage of control cells. * *p* < 0.05, ** *p* < 0.01, *** *p* < 0.001

Acyclic laxaphycins obtained from *A*. *torulosa* (compounds **3** and **4**) did not display cytotoxicity at any of the concentrations tested (Figure 5a,f). Laxaphycin **3** significantly augmented cell metabolic activity at 0.1 and 1 µM and produced a decrease in ∆Ψ_m_ (Figure 5b,c). This compound also reduced ROS and ATP levels at the highest concentrations (Figure 5d,e). Interestingly, the reduction in ATP was observed at 6 h, but it was recovered at 24 h, reaching levels of control cells. Compound **4** presented similar results, depolarizing the mitochondrial membrane at 10 µM, and diminishing ROS release and ATP levels at 6 h (Figure 5h–j).

**Figure 6:**
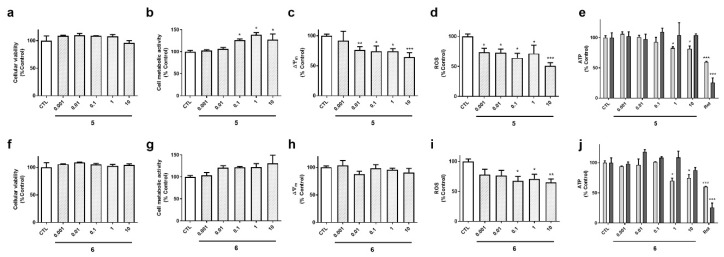
Effect of acyclic compounds obtained from *S. striatus* on cell viability and mitochondrial function in human neuroblastoma cells. SH-SY5Y cells were treated with compounds **5** (**a**–**e**) and **6** (**f**–**j**) for 24 h and their effects on cell viability, metabolic activity, ∆Ψ_m_, ROS and ATP levels were assessed with LDH assay, MTT test, TMRM dye, carboxy-H_2_DCFDA and a commercial kit, respectively. ATP assay was performed at 6 h (light bars) and 24 h (dark bars). Concentration expressed in µM. Mean ± SEM of three experiments performed by triplicate. Values are presented as percentage of control cells. * *p* < 0.05, ** *p* < 0.01, *** *p* < 0.01

The biological activities of compounds **5** and **6**, isolated from the gastropod *S. striatus*, were also determined (Figure 6). These laxaphycins did not show effects on cell survival (Figure 6a,f). Compound **5** increased cell metabolic activity, depolarized the mitochondria and reduced ROS levels at the highest concentrations (Figure 6b–d). With respect to ATP, its levels were reduced by **5** after a 6 h incubation, and recovered at 24 h (Figure 6 e). Laxaphycin **6** only affected to ROS and ATP levels (Figure 6i–j), it decreased ROS release and reduced ATP content at 6 h, recovering it after 24 h, as happened with the other acyclic laxaphycins.

### 2.4. Cyclic Laxaphycin-B Peptides Induce Apoptosis in SH-SY5Y Cells

**Figure 7:**
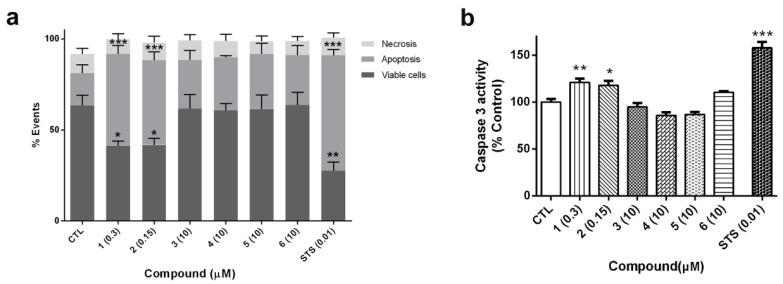
Cyclic laxaphycin B peptides induce apoptotic cell death in neuroblastoma cells. (**a**) Cells were treated for 24 h with compounds and stained with Annexin V-FITC and propidium iodide, and the fluorescence was analyzed by flow cytometry. Staurosporine (STS) was used as positive control. Apoptosis include early and late apoptotic cells. Data are expressed as percentage of total cells. (**b**) The activity of caspase 3 after treatment with laxaphycins during 24 h was assessed with a commercial kit. Results are expressed as percentage of control cells. Mean ± SEM of three independent experiments. * *p* < 0.05, ** *p* < 0.01, *** *p* < 0.001

In view of the cytotoxicity displayed by cyclic laxaphycins **1** and **2** in LDH and MTT assays, the type of cell death produced by compounds was determined. SH-SY5Y cells were treated with compounds for 24 h. Laxaphycins **1** and **2** were used at IC_50_ values obtained from MTT assay (0.3 and 0.15 µM, respectively). Otherwise, acyclic laxaphycins **3**, **4**, **5** and **6** were tested at 10 µM. Cells were co-stained with Annexin V-Fluorescein (FITC) and propidium iodide (PI) and the fluorescence was analyzed by flow cytometry (Figure 7a). The percentages of apoptotic cells, including early apoptotic cells (Annexin V positive and PI negative) and late apoptotic cells (Annexin V positive and PI positive), and necrotic cells (Annexin V negative and PI positive) were calculated. Cells treated with compounds **1** and **2** produced a significant decrease in cell survival (around 60% of control cells). The values of Annexin-V-positive cells in these treatments confirmed that cyclic B-type laxaphycins triggered an apoptotic process. The addition of **1** and **2** produced a 50.4% ± 4.8% (*p* < 0.001) and 46.4% ± 4.8% (*p* < 0.001) of apoptotic cells, respectively. As expected, treatment with staurosporine (STS) also generated apoptosis in neuroblastoma cells (63.3% ± 3.2%, *p* < 0.001). On the other hand, acyclic laxaphycins did not show any significant effect on cell viability, agreeing with our previous results.

To confirm the results obtained with flow cytometry, the activity of caspase 3 was analyzed. This enzyme is an executioner caspase, involved both in intrinsic and extrinsic apoptosis, which targets several apoptotic substrates and initiates a cascade of events that results in cell death [28]. SH-SY5Y cells were treated with laxaphycins for 24 h at the same concentrations used in flow cytometry assays and caspase 3 activity was evaluated in cell lysates. As Figure 7b shows, compounds **1** (0.3 µM) and **2** (0.15 µM) produced a significant increase in the activity of the executioner caspase (about 20% of control cells). Once again, acyclic laxaphycins did not affect to caspase 3 activity, which is in agreement with the results of flow cytometry. STS addition also augmented the enzymatic activity of the caspase (157.8% ± 6%, *p* < 0.001).

### 2.5. Acyclic B-Type Laxaphycins Affect to Autophagy in Human Neuroblastoma Cells

**Figure 8:**
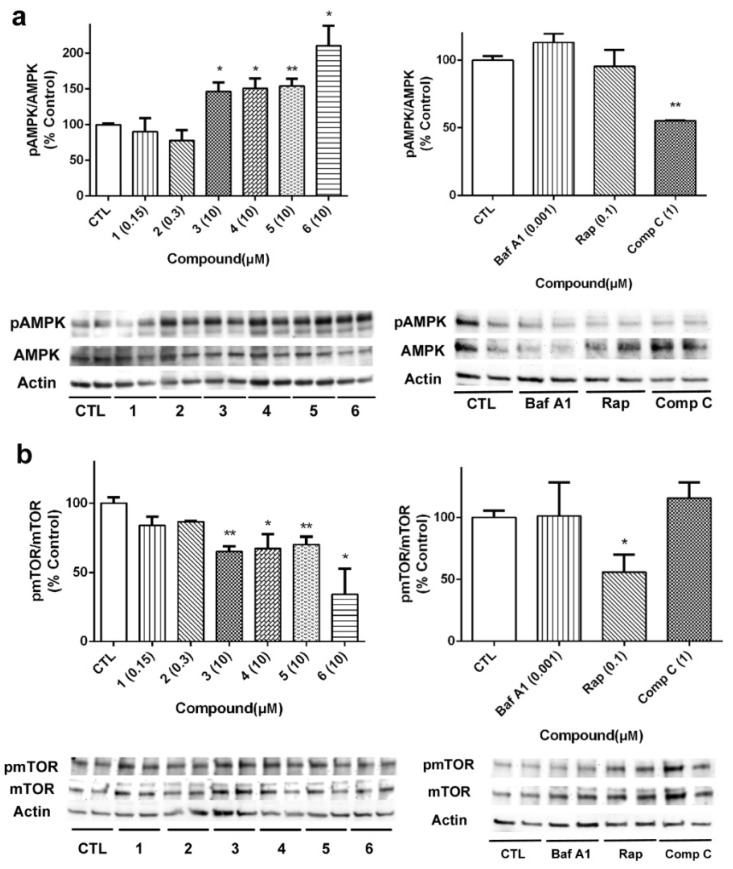
Analysis of autophagy initiation-related proteins in SH-SY5Y cells treated with B-type laxaphycins. Compounds were added for 24 h and the protein expression of (**a**) AMPK and (**b**) mTOR was determined by Western blot. Left panels show the results of compounds and right panels present the results of the positive controls bafilomycin A1 (Baf A1), rapamycin (Rap) and compound C (Comp C). The activation of AMPK and mTOR was analyzed as the ratio between phosphorylated/total protein levels. Protein expression levels are normalized by β-actin. Values are mean ± SEM of three replicates performed in duplicate, expressed as percentage of control cells. * *p* < 0.05, ** *p* < 0.01

Considering the results obtained with acyclic B-type laxaphycins in the mitochondrial function and our previous work with A-type laxaphycins [14], we decided to evaluate the effect of these compounds on the autophagic flux. With this purpose, SH-SY5Y cells were treated with laxaphycins B for 24 h, and the expression of proteins involved in autophagy was determined by Western blot. Bafilomycin A1 (Baf A1), an inhibitor of the fusion of autophagosomes and lysosomes, rapamycin (Rap), a mTOR inhibitor, and compound C (Comp C), an AMPK inhibitor, were used as positive controls in these assays [29]. Firstly, the effect of compounds on AMPK activation was determined, since this kinase is known to activate autophagy under circumstances of energy depletion [30]. Acyclic peptides produced a significant increase in the activation of AMPK, with compound **6** being the most active (Figure 8a). As expected, Comp C at 1 µM inhibited kinase activity. Then, we analyzed the expression of mTOR, considered the master regulator of the autophagic process. Cyclic laxaphycins **1** and **2** did not affect to the protein expression, whereas the acyclic peptides produced a significant reduction in its activation (Figure 8b). Treatment with 0.1 µM Rap also reduced mTOR activation.

**Figure 9:**
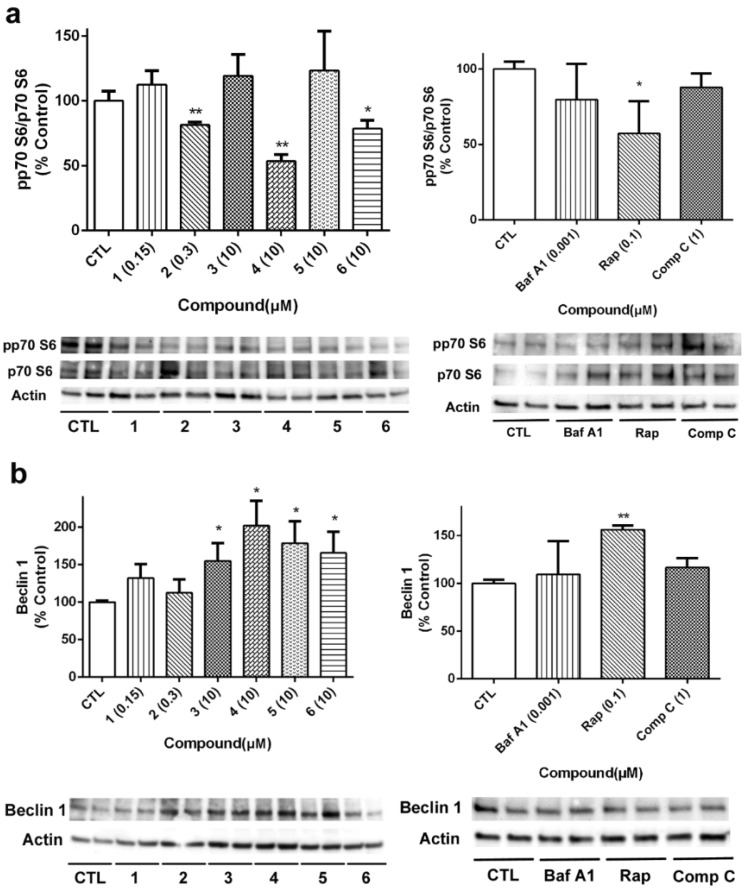
Analysis of p70 S6 kinase and Beclin 1 expression in neuroblastoma cells after treatment with B-type laxaphycins. The protein expression of (**a**) p70 S6 and (**b**) beclin 1 was determined by Western blot. Left panels show the results of compounds and right panels present the results of the positive controls bafilomycin A1 (Baf A1), rapamycin (Rap) and compound C (Comp C). The activation of the kinase was analyzed as the ratio between phosphorylated/total protein levels. Protein expression levels are normalized by β-actin. Values are mean ± SEM of three replicates performed in duplicate, expressed as percentage of control cells. * *p* < 0.05, ** *p* < 0.01

The activation of p70 S6 kinase, an mTOR downstream target, was also determined (Figure 9a). The cyclic peptide **2** and the acyclic laxaphycins **4** and **6** diminished p70 S6 phosphorylation, reaching percentages among 53.5%–78.6% of untreated cells. Next, we analyzed the expression of beclin 1, a component of the complex that starts autophagosome formation [31]. As can be seen in Figure 9b, acyclic compounds **3**, **4**, **5** and **6** generated an increase in beclin1 expression. Treatment with the autophagy activator Rap also augmented its expression.

**Figure 10:**
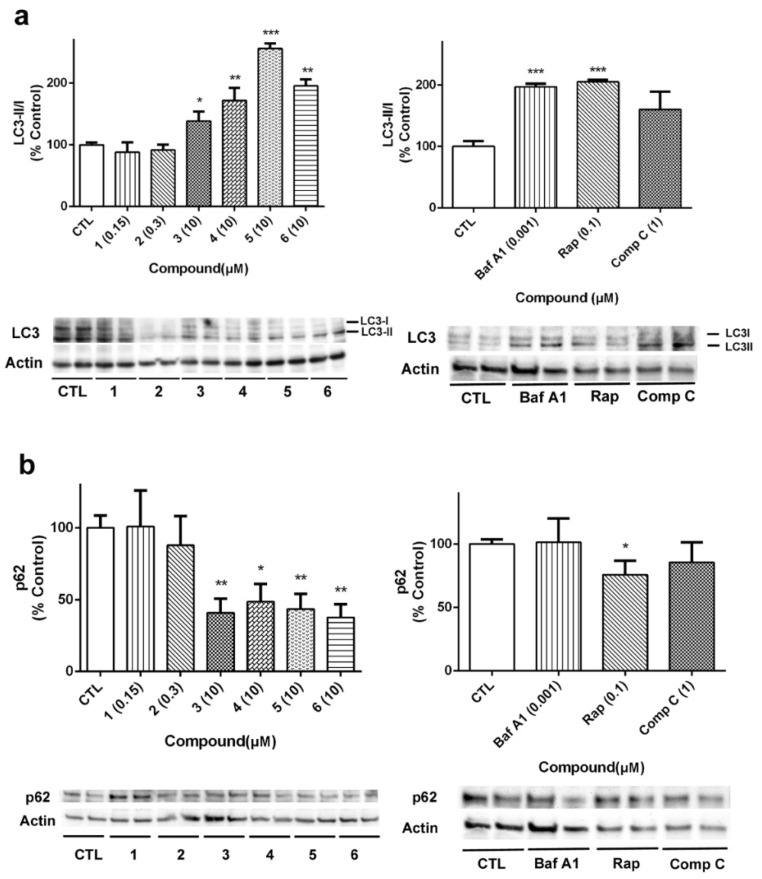
Determination of the effect of laxaphycins B on LC3 and p62 expression. SH-SY5Y cells were treated with compounds for 24 h and protein expression was analysed by Western blot. (**a**) LC3 expression, calculated as the ratio between LC3II (the lipidated form) and LC3I (the soluble form). (**b**) p62 expression. Left panels show the results of compounds and right panels present the results of the positive controls bafilomycin A1 (Baf A1), rapamycin (Rap) and compound C (Comp C). Protein expression levels are normalized by β-actin. Values are mean ± SEM of three replicates performed in duplicate, expressed as percentage of control cells. * *p* < 0.05, ** *p* < 0.01, *** *p* < 0.001

Beclin 1 is implicated in other cellular mechanisms, such as apoptosis, so its quantification must be complemented with other proteins involved in autophagy [29]. In our case, the expression of LC3 and p62 was analyzed to further confirm the effects of laxaphycins over autophagy (Figure 10). As Figure 10a shows, LC3II/I ratio was increased when acyclic laxaphycins were added to neuroblastoma cells, confirming our previous results. Treatment with Baf A1 and Rap also increased LC3II/I ratio. With regard to p62, whose degradation is related to autophagy, treatment with acyclic laxaphycins produced a significant decrease in its expression, with levels between 37.7–48.6% of untreated cells, a greater degradation than that produced by Rap (Figure 10b). In summary, these results suggest that acyclic laxaphycins B affect to the autophagic flux in human neuroblastoma cells, which is probably mediated by their effects on mitochondria.

## 3. Discussion

Laxaphycins are a large family of lipopeptides synthetized through a hybrid PKS/NRPS pathway by different cyanobacteria species. These peptides, selected by evolution in both freshwater and oceanic cyanobacteria, may confer to these primary producers an ecological advantage.

Laxaphycins are divided into two sub-families, laxaphycin A-type and laxaphycin B-type peptides. Laxaphycin B-type members such as laxaphycins B, B2, and B3 have presented antifungal, antimicrobial and cytotoxic activities [9,10,11,12], while laxaphycin A-type compounds have shown only weak cytotoxicity [10,12,14].

Here we describe the complete structural elucidation of four acyclic B-type laxaphycins, two of them (**3** and **4** with ring opening) corresponding to the acyclic analogues of laxaphycins B and B3, isolated from the cyanobacteria *A. torulosa*, and the other two (**5** and **6** with ring opening and deletion of two amino-acids) are diet-derived compounds isolated from the herbivorous gastropod *S. striatus* that feeds on the cyanobacteria. It is not unlikely that these acyclolaxaphycins B ensued from an adaptative biotransformation mechanism from a specific herbivorous species [32]. Cyclic lipopeptides are relatively widespread in cyanobacteria, but such biotransformation, ring opening and amino-acids deletion, had never been described.

Moreover, this study is the first description of the effects on mitochondria and autophagy produced by acyclic B-type laxaphycins, and also confirms that the toxic effects of cyclic laxaphycins B are mediated by an apoptotic process. The cytotoxicity of cyclic laxaphycins had been tested in previous works in which laxaphycin B showed cytotoxic effects against a panel of cancer cell lines (IC_50_ < 2 µM), and both compounds (laxaphycins B and B3) had displayed toxicity towards drug-sensitive and multidrug-resistant tumor cell lines (IC_50_ ≈ 1 µM) [9]. Our results in neuroblastoma cells are in agreement with these previous assays, with IC_50_ values of 1.8 and 0.8 µM for laxaphycins B and B3, respectively. A previous work had hypothesized that laxaphycin B toxicity could be mediated by the inhibition of topoisomerase II [10]. The inhibition of this enzyme causes DNA disorders that enhance apoptotic cell death [33]. In the current work, we provide new data that support the triggering of an apoptotic process by laxaphycins B and B3, as indicated by Annexin V staining and caspase 3 activation. The results obtained in p70 S6 kinase, whose inhibition is involved both in autophagy and apoptosis activation [34,35], suggest a different mechanism of action of these cyclic peptides, since only laxaphycin B3 reduced the phosphorylation of this enzyme. On the other hand, the biological activities of acyclic B-type laxaphycins had not been tested so far. Our results suggest that these compounds affect the mitochondrial function, producing a decrease in ATP levels, which may lead to the activation of AMPK. Moreover, acyclic laxaphycins have an impact on the autophagic flux, as evidenced by their effect on the expression of proteins related to this cellular event. Further experiments will help us to clarify if the effect on autophagy is being produced by the activation of AMPK.

AMPK is activated when ATP levels decrease, and maintains energy homeostasis through the inhibition of energy-consuming processes, such as protein and lipid biosynthesis, and the activation of ATP-producing pathways, such as glucose metabolism and mitochondrial biogenesis. Moreover, when an energy depletion occurs, AMPK triggers the autophagic flux, since this catabolic process provides energy and substrates for the synthesis of new biomolecules [22]. Thus, increasing AMPK activity will be a good strategy to increase cellular energy and avoid energetic failure in vulnerable cells. There are several examples of indirect AMPK activators that act through the decrease of ATP levels. Natural compounds such as quercetin, resveratrol, genistein and curcumin activate the kinase by targeting components of the oxidative phosphorylation and increasing the AMP/ATP ratio [36]. However, the most studied AMPK activator is the biguanide metformin, in clinical use for the treatment of Type 2 diabetes mellitus. Metformin inhibits the complex I of mitochondrial respiratory chain, reducing the proton gradient, the production of ROS and ATP levels. This leads to the activation of AMPK and the subsequent increase in glucose uptake [37]. Along with their use as antidiabetic agents, the therapeutic application of AMPK activators has been expanded to the treatment of cancer, inflammation and neurodegeneration [38].

The phosphorylation of mTOR, considered the main regulator of autophagy, was also inhibited by treatment with acyclic laxaphycins B. mTOR plays a central role in integrating growth signals and controlling their physiological effects at a cellular level. Its activation upregulates anabolic processes such as synthesis of proteins, lipids and nucleotides, and downregulates catabolic mechanisms such as autophagy. However, mTOR hyperactivation is linked to several diseases such as cancer, since the kinase promotes tumor growth and proliferation, as well as diabetes, in which it contributes to insulin resistance [39]. Currently, mTOR inhibitors are in clinical use as immunosuppressants and anti-cancer drugs, and due to crucial role of the kinase in many illnesses, it is expected that mTOR inhibitors may have a broader application for other diseases [40,41].

Along with these illnesses, there is substantial evidence of autophagy dysregulation in neurodegenerative diseases, such as alterations in mTOR and beclin 1 expression, and accumulation of defective autophagosomes [42]. In this context, the activation of the autophagic flux has emerged as a therapeutic approach for these pathologies, because it represents a major pathway for clearance of aggregated proteins and damaged organelles. Several compounds such as metformin or Rap have shown promising effects in vivo [42], and the activation of mitophagy has been recently related to a reduction in cognitive deficits in Alzheimer’s disease models [43]. Therefore, the stimulation of autophagy maybe a good strategy to face neurodegeneration. However, the use of activators must be handled carefully, as an excessive autophagy can lead to the destruction of essential cellular machinery [44]. In addition, intervention in early phases of the diseases would be more favorable than in an advanced state [21]. The results obtained with acyclic B-type laxaphycins in the human neuroblastoma cell line SH-SY5Y, which maintains certain characteristics of neuronal cells [23], could be used as a starting point to analyse their neuroprotective effects. In this sense it would be interesting to study the ability of laxaphycins to cross the blood brain barrier (BBB). There are several examples of non-ribosomal peptides capable of penetrating the BBB, such as polymyxins [45] and some cyanotoxins [46], which can reach the brain through specific peptide transporters. Moreover, the lipidic nature of laxaphycins could allow the compounds to cross the BBB, as lipid solubility is a crucial factor that facilitates transport across the barrier [47].

On the other hand, the data obtained in this work show a clear correlation among the chemical structure and the biological activities of laxaphycins B. Cyclic laxaphycins produce apoptotic cell death and exhibit a greater cytotoxicity than their acyclic analogues. Also, the results obtained in p70 S6 kinase with the cyclic laxaphycin B3 and their acyclic analogs suggest that the presence of an OH group in R is a key structural feature to the inhibition of this enzyme. Due to the supply problem associated with marine compounds, the chemical synthesis of B-type laxaphycins would be a good strategy for their pharmacological use. Laxaphycin B total synthesis has been previously published [48], as well as the synthesis of two simplified analogues of the parent compound [7], which opens a door for future synthesis of acyclic laxaphycins, which will help to better understand the structure-activity relationship of B-type laxaphycins.

In summary, this work provides new data that confirm the pro-apoptotic effects produced by cyclic laxaphycins B, and describes for first time the complete structural elucidation of four B-type acyclic laxaphycins and their biological activities. The acyclic peptides affect mitochondrial function and have an effect on the expression of key proteins involved in autophagic flux, suggesting an involvement of the compounds in the activation of this mechanism. Further experiments will help to clarify the mechanism of action of the B-type laxaphycins and the structural requirements for these activities.

## 4. Materials and Methods

### 4.1. Chemicals and Solutions

EnzCheck^®^ Caspase-3 Assay Kit, TMRM, 5-(and-6)-carboxy-2′, 7′-dichlorodihydrofluorescein diacetate (carboxy-H_2_DCFDA), Pierce ^TM^ Protease Inhibitor Mini Tablets and Pierce^TM^ Phosphatase Inhibitor Mini Tablets were purchased from Thermo Fisher Scientific (Waltham, MA, USA). Annexin V-FITC Apoptosis Detection Kit was obtained from Immunostep (Salamanca, Spain). Rap, Comp C and Baf A1 were obtained from Abcam (Cambridge, UK). Other chemicals were reagent grade and were purchased from Sigma-Aldrich (Madrid, Spain).

### 4.2. Organism Collection

The cyanobacterium *Anabaena torulosa*, as well as the herbivorous gastropod *Stylocheilus striatus,* were collected by SCUBA diving at a depth of 1–5 m in Moorea Atoll, French Polynesia (S 17°29′22″, W 149°54′17″) in Pacific Ocean. The cyanobacterium and the gastropod samples were sealed independently underwater in a bag with seawater and then frozen and freeze-dried.

### 4.3. HPLC and LC-MS Analyses

HPLC-PDA-ELSD analyses were performed with a Waters Alliance HPLC system (W 2695) coupled to a photodiodes array detector (PDA Waters 2998) and an evaporative light scattering detector (Waters ELSD 2424). The analyses were performed on a reversed-phase column (Thermo Hypersil Gold C-18, 150 × 2.1 mm, 3 µm) employing a gradient of 10% to 100% CH_3_CN over 40 min followed by 25 min at 100% CH_3_CN (all solvents buffered with 0.1% formic acid) with a flow rate of 0.3 mL/min. Semi-preparative HPLC purifications were performed on a binary HPLC pump system Waters 1525 with a dual λ absorbance detector Waters 2487, equipped with a reverse phase column (Interchim UP5ODB.25M, 250 × 10 mm, 5 µm) using isocratic elution (H_2_O-CH_3_CN) at a flow rate of 3 mL/min.

### 4.4. Compound Isolation and Purification

Six hundred grams of freeze-dried *A*. cf *torulosa* and 4.24 g of *S. striatus* (6 specimens) were extracted, separately but in the same manner, with a mixture of CH_3_OH-CH_2_Cl_2_ (1:1) and sonicated during 10 minutes to yield two organic extracts after evaporation under reduced pressure. Then the two crude extracts (38 g and 4 g, respectively) were subjected to flash RP18 silica gel column eluted with H_2_0 (A), H_2_O-CH_3_CN (20:80) (B) and CH_3_OH-CH_2_Cl_2_ (80:20) (C) to afford 3 fractions (A, B and C). Fractions B, from *A. torulosa* and *S. Striatus*, were fractioned, in turn, with flash chromatography and RP18 silica gel column with a gradient of H_2_O-CH_3_CN to give 7 sub-fractions. Fraction B4 from the organic extract of *S. striatus* subjected to HPLC purification (Phenomenex Gemini C6-phenyl, 110Å, 250 × 10 mm, 5 µm) and eluted with 28% CH_3_CN in H2O with 0.1% formic acid at a flow rate of 4 mL/min, gave compounds **5** (2.5 mg) and **6** (5 mg). Laxaphycin A was found in sub-fraction B5, but none of the already described laxaphycins B or B3 could be detected. Fraction B4 from the organic extract of *A. torulosa* was eluted with 38% CH_3_CN and gave compounds **3** (3 mg) and **4** (4 mg). Laxaphycins A, B (**1**) and B3 (**2**) were found respectively in sub-fraction B6 and B5.

### 4.5. Mass Spectrometry and NMR Spectroscopy

LC-MS analyses were carried out using a Thermo Fisher Scientific LC-MS device, Accela HPLC coupled to an LCQ Fleet equipped with an electrospray ionization source and a 3D ion-trap analyzer. High-resolution ESI mass spectra were obtained on a Bruker Thermo Scientific Q-Tof Maxis mass spectrometer using electrospray ionization in positive mode. Compounds were solubilized in MeOH at 1 µg/mL and infused in mass spectrometer (collision energy: 50 eV).

1D-NMR and 2D-NMR experiments were acquired on a Brucker Avance 800 spectrometer equipped with a cryogenic probe (5 mm), all compounds solubilized in DMSO-*d*6 (500 µL) at 303 K. All chemical shifts were calibrated on the residual solvent peak (DMSO-*d*6, 2.50 ppm (^1^H) and 39.5 ppm (^13^C). The chemical shifts (δ), reported in parts per million (ppm) are referenced relatively to TMS.

### 4.6. Advanced Marfey’s Analyses

The Marfey’s analyses were carried out on compounds **3**, **4**, **5** and **6**. Approximately 0.3 mg of each compound were hydrolyzed with 1 mL of 6 N HCl for 20 h at 110 °C in sealed glass vials. The cooled hydrolysate mixtures were evaporated to dryness and traces of HCl were removed from the reaction mixtures by repeated evaporation. Each hydrolysate mixture was dissolved in H_2_O (100 µL). 110 µL of acetone, 20 µL of 1 N NaHCO_3_ and 20 µL of 1% L or D/L FDLA (1-fluoro-2,4-dinitrophenyl-5-L-leucinamide) in acetone were added to each 50 µL aliquot. The mixtures were then heated to 40 °C for 1 h. The cooled solutions were neutralized with 1 N HCl (20 μL), and then dried in vacuo. The residues were dissolved in 1:1 CH_3_CN-H_2_O and then analyzed by LC-MS. LC-MS analyses were performed on a reversed-phase column (Thermo Hypersil Gold C-18, 150 × 2.1 mm, 3 μm) with two linear gradients: (1) from 20% CH_3_CN-80% 0.01 M formic acid to 60% CH_3_CN-40% 0.01 M formic acid at 0.3 mL/min over 70 min and (2) from 10% CH_3_CN-90% 0.01 M formic acid to 50% CH_3_CN-50% 0.01 M formic acid at 0.3 mL/min over 70 min, then to 80% CH_3_CN-20% over 10 min. The configuration of the α carbon for each residue can be assigned in accordance with the elution order of the D- and L-FDLA derivatives [24,25]: amino acids for which the D-FDLA analogue elutes first have a D configuration, whereas those for which the L-FDLA analogue elutes first have an L configuration. Furthermore, the hydrolysates were compared to that of laxaphycin B.

### 4.7. Cell Culture

SH-SY5Y human neuroblastoma cell line was obtained from American Type Culture Collection (ATCC), number CRL2266. Cells were cultured in Dulbecco´s modified Eagle´s medium: Nutrient Mix F-12 (DMEM/F-12) with 10% fetal bovine serum (FBS), 1% glutamax, 100 U/mL penicillin and 100 µg/mL streptomycin. Cells were maintained at 37 °C in a humidified atmosphere of 5% CO_2_ and 95% air and dissociated weekly using 0.05% trypsin/EDTA. All the reagents were obtained from Thermo Fischer Scientific.

### 4.8. Cytotoxicity Assay

Cell viability was assessed with the LDH test [14]. Cells were seeded in 96-well plates and exposed to different compound concentrations (0.001–10 µM). Cells were incubated with compounds at 37 °C in humidified 5% CO_2_/95% air atmosphere for 24 h. Quillaja bark saponin was used as cellular death control. Then, cell medium was collected and LDH release was evaluated. Pierce^TM^ LDH-Cytotoxicity Assay Kit (Thermo Fisher Scientific) was used for LDH determination. Absorbance was measured at 490 nm to determine LDH release to the medium. Experiments were carried out in triplicate at least three independent times.

### 4.9. Metabolic Activity Evaluation

The effect of laxaphycins on cell metabolic activity was evaluated by MTT (3-(4, 5-dimethyl thiazol-2-yl)-2, 5-diphenyl tetrazolium bromide) assay [27,49]. SH-SY5Y cells were cultured in 96-well plates and treated with compounds at concentrations ranging from 0.001 to 10 µM for 24 h. Next, cells were rinsed three times and incubated for 1 h with MTT (500 µg/mL) dissolved in saline buffer. MTT excess was washed and cells were disaggregated with 5% sodium dodecyl sulfate. Absorbance of the colored formazan salt was measured at 595 nm in a spectrophotometer plate reader. Cell death control signal was subtracted from the other data.

### 4.10. Mitochondrial Membrane Potential Measurement

∆Ψ_m_ was evaluated with TMRM assay as previously described [49]. SH-SY5Y cells were seeded in 96-well plates at 5 × 10^4^ cells per well. After 24 h, cells were treated with compounds (0.001–10 µM) for 24 h. Next, cells were washed twice with saline solution and incubated with 1 µM TMRM for 30 min. Then human neuroblastoma cells were solubilized with 50% DMSO–50% water. Fluorescence values were measured at 535 nm excitation, 590 nm emission with a plate reader. At least three independent replicates were performed in triplicate.

### 4.11. Evaluation of Reactive Oxygen Species and ATP Levels

ROS production was assessed with the fluorescence dye carboxy-H_2_DCFDA (5-(and-6)-carboxy-2′,7′-dichlorodihydrofluorescein diacetate) [49]. SH-SY5Y cells were seeded in 96-well plates and allowed to grow for 24 h. Then, cells were treated with laxaphycins (0.001–10 µM) for 24 h and ROS levels were measured. SH-SY5Y cells were washed twice with serum-free medium and loaded with 20 µM carboxy-H_2_DCFDA. Next, the plate was incubated for 1 h at 37 °C. After this time, phosphate buffered saline (PBS) was added to each well during 30 min. The fluorescence was read at 495 nm excitation and 527 nm emission. Experiments were carried out at least three times.

ATP levels were determined with the Luminescent ATP Detection Kit (Abcam), following manufacturer´s instructions. Briefly, human neuroblastoma cells were cultured in 96-well plates at 5 × 10^4^ cells per well. Cells were treated with compounds as described above during 6 and 24 h, Then, cells were lysed and 50 µL of substrate solution were added to each well. The plate was incubated for 5 min, and the luminescence was measured in a plate reader. Rot at 1 µM was used as positive control.

### 4.12. Flow Cytometry Analysis

The Annexin V-FITC Apoptosis Detection Kit was used to determine the cell death produced by compounds as previously described [50]. SH-SY5Y cells were seeded in 12-well plates at 1 × 10^6^ per well and incubated for 24 h with laxaphycins. Then, cells were washed with PBS and resuspended in Annexin binding buffer containing Annexin V-FITC and PI. Cells were incubated for 15 min at room temperature, resuspended in PBS and filtered. The fluorescence was determined by flow cytometry using the ImageStreamMKII (Amnis Corporation, Luminex Corp, Austin, TX, USA). 10,000 events were analyzed with IDEAS Application 6.0 software (Amnis Corporation, Luminex Corp, Austin, TX, USA). STS (Sigma Aldrich) at 0.01 µM was used as control in this assay.

### 4.13. Evaluation of Caspase 3 Activity

The detection of caspase 3 activity was carried out with the EnzCheck^®^ Caspase-3 Assay Kit. Neuroblastoma cells were cultured in 12-well plates at 1 × 10^6^ cells per well and treated with compounds. After 24 h, cells were lysed and an aliquot was collected to quantify protein concentration by the Bradford method. Then, the assay was performed following manufacturer’s instructions. Briefly, 50 µL of each sample were mixed with equal volume of Z-DEVD-AMC substrate and incubated for 30 min at room temperature. Then, fluorescence at 342 nm excitation and 441 nm emission was measured. Caspase 3 activity data were corrected by protein concentration values. STS (0.01 µM) was used as positive control.

### 4.14. Western Blotting

SH-SY5Y cells were seeded in 12-well plates and treated with laxaphycins for 24 h. Next, cells were washed with PBS and lysis buffer (50mM Tris HCl, 150 mM NaCl, 1mM EDTA and 1% Triton x-100, supplemented with a complete phosphatase/protease inhibitor cocktail) was added to each well. Cells were scrapped, sonicated and centrifuged at 13,000 rpm at 4 °C for 20 min.

For p62 quantification, cells were lysed as previously described, with modifications [14]. A hypotonic buffer (20 mM Tris-HCl, pH 7.4, 10 mM NaCl, 3 mM MgCl_2_, containing a complete phosphatase/protease inhibitor cocktail) was added to each well. Then, cells were incubated for 15 min on ice, sonicated and centrifuged at 3000 rpm at 4 °C for 15 min.

In both cases, the supernatant was collected as the cytosolic fraction and quantified with the Bradford method. Samples containing 15 µg were used for electrophoresis, resolved in 4–20% sodium dodecyl sulphate polyacrylamide gel (Biorad, Madrid, Spain). Proteins were transferred to PVDF membranes (Merck Millipore) with a Trans-Blot^®^ semi-dry transfer cell (Biorad). Membrane blocking and antibody incubation was performed in Snap i.d. system (Merck Millipore). Protein bands were detected with Supersignal West Pico Luminiscent Substrate and Supersignal West Femto Maximum Sensitivity Substrate (Thermo Fisher Scientific). Chemiluminiscence was determined with Diversity GeneSnap system and software (Syngene) [51]. LC3 II/I was detected with the primary antibody anti-LC3 II/I (1:1000, Abcam), the primary antibody anti-pmTOR (Ser2448) (1:1000, Merck Millipore) was used to recognize phospho-mTOR and the total levels of the kinase were determined with anti-mTOR antibody (1:10000, Abcam), beclin-1 was detected with anti-beclin-1 primary antibody (1:1000, Merck Millipore), p62 was quantified with anti-p62 antibody (1:1000, Merck Millipore), anti-phospho-p70 S6 kinase (Thr389) (1:1000, Merck Millipore) was used to detect phospho- p70 and the total levels were quantified using anti-p70 S6 kinase antibody (1:1000, Merck Millipore), anti-phospho-AMPK (Thr172) (1:500, Merck Millipore) was used to recognize the phosphorylated state of the kinase, and the total expression of the enzyme was detected with anti-AMPK antibody (1:500, Merck Millipore). Protein levels were normalized using β-Actin (1:20,000, Millipore). Baf A1 (0.001 µM) [29], Rap (0.1 µM) [52] and Comp C (1 µM) [53] were used as controls for autophagic flux measurements. All the experiments were performed at least three times in duplicate.

### 4.15. Statistical Analysis

Data are presented as mean ± SEM. Statistical differences were evaluated by one-way ANOVA or Student´s *t*-tests with Graph Pad Prism 6 software. Statistical significance was considered at *p* < 0.05.

## Figures and Tables

**Table 1:** NMR spectroscopic data for [des-(Ala^4^-Hle^5^)] acyclolaxaphycin B (**5**) and [des-(Ala^4^-Hle^5^)] acyclolaxaphycin B3 (**6**) (303 K) in DMSO-d6.

	[des-(Ala^4^-Hle^5^)] Acyclolaxaphycin B (5)			[des-(Ala^4^-Hle_5_)] Acyclolaxaphycin B3 (6)		
		^13^C	^1^H		^13^C	^1^H
		δ (ppm)	δ (ppm)		δ (ppm)	δ (ppm)
**Gln^1^**	NH_2_			NH		
	CαH	53.01	4.50	CαH	53.15	4.51
	CβH_2_	29.95	1.88	CβH_2_	29.91	1.90
	Cγ H_2_	28.76	2.20	Cγ H_2_	29.12	2.20
	CON	174.24		CONH	174.27	
	NH_2_		6.74/7.30			
	CO	172.85		CO	172.90	
**N-MeILe^2^**	NCH_3_	29.53	2.88	NCH_3_	29.60	2.88
	CαH	59.75	4.73	CαH	59.68	4.73
	CβH	31.00	1.94	CβH	31.12	1.92
	CγH_2_	23.59	0.92/1.31	CγH_2_	24.02	0.89/1.29
	Cγ’H_3_	15.16	0.78	Cγ’H_3_	15.20	0.78
	CδH_3_	10.32	0.81	CδH_3_	10.41	0.80
	CO	169.71		CO	169.76	
**Has^3^**	NH		7.49	NH		7.52
	CαH	55.12	4.62	CαH	55.36	4.63
	CβH	70.84	4.37	CβH	70.91	4.37
	OH			OH		
	CONH_2_	173.26		CONH_2_	173.29	
	NH_2_		7.27/7.35	NH_2_		7.28/7.35
	CO	168.73		CO	168.79	
**Thr^4^**	NH		7.50	NH		7.46
	CαH	54.99	4.57	CαH	55.25	4.56
	CβH	66.37	3.99	CβH	66.40	3.98
	OH			OH		
	CγH_3_	18.34	1.01	CγH_3_	18.42	1.01
	CO	168.95		CO	169.02	
**Pro^5^/Hyp^5^**	CαH	59.89	4.39	CαH	59.23	4.40
	CβH_2_	28.91	1.84/2.03	CβH_2_	37.69	1.87/2.04
	CγH(_2_)	24.07	1.81/1.89	CγH	68.41	4.29
	OH			OH		
	CδH_2_	47.36	3.64/3.76	CδH_2_	55.78	3.59/3.78
	CO	170.38		CO	171.45	
**Leu^6^**	NH		7.89	NH		7.88
	CαH	51.63	4.28	CαH	51.61	4.27
	CβH_2_	40.15	1.46/1.54	CβH_2_	40.38	1.48/1.55
	CγH	23.89	1.59	CγH	24.03	1.60
	CδH_3_	22.91	0.85	CδH_3_	22.96	0.86
	Cδ’H_3_	21.26	0.83	Cδ’H_3_	21.34	0.83
	CO	172.12		CO	172.27	
**Thr^7^**	NH		7.80	NH		7.75
	CαH	58.50	4.07	CαH	58.47	4.09
	CβH	66.37	3.99	CβH	66.43	3.98
	OH			OH		
	CγH_3_	19.62	0.99	CγH_3_	19.66	0.99
	CO	169.17		CO	169.74	
**β-Ade^8^**	NH		7.56	NH		7.57
	CαH_2_	39.92	2.36	CαH_2_	40.29	2.36
	CβH	46.30	4.00	CβH	46.35	4.02
	CγH_2_	33.19	1.35/1.41	CγH_2_	33.31	1.34/1.40
	CδH_2_	28.72	1.20	CδH_2_	28.86	1.20
	CεH_2_	28.60	1.20	CεH_2_	28.75	1.20
	CζH_2_	25.37	1.20	CζH_2_	25.41	1.20
	Cη H_2_	31.20	1.20	Cη H_2_	31.26	1.20
	Cθ H_2_	22.01	1.24	Cθ H_2_	22.15	1.25
	CιH_3_	13.84	0.85	CιH_3_	13.97	0.84
	CO	170.23		CO	170.45	
**Val^9^**	NH		7.89	NH		7.90
	CαH	57.73	4.23	CαH	57.73	4.26
	CβH_2_	29.88	2.03	CβH_2_	30.35	2.03
	CγH_3_	19.28	0.82	CγH_3_	19.25	0.82
	Cγ’H_3_	17.67	0.81	Cγ’H_3_	17.77	0.81
	CO	170.57		CO	170.85	
**Hle^10^**	NH		7.55	NH		7.59
	CαH	54.87	4.27	Cα	54.85	4.30
	CβH	75.89	3.52	CβH	76.02	3.51
	OH			OH		
	CγH	30.52	1.53	CγH	30.73	1.51
	CδH_3_	19.22	0.82	CδH_3_	19.119	0.88
	Cδ’H_3_	19.16	0.77	Cδ’H_3_	19.12	0.76
	CO	172.02		CO	172.09	

## Supplementary Materials

The following are available online at https://www.mdpi.com/1660-3397/18/7/364/s1, Figure S1: 1H-NMR spectrum of [des-(Ala4-Hle5)] acyclolaxaphycin B (5) in DMSO (303K), Figure S2: 13C-NMR spectrum of [des-(Ala4-Hle5)] acyclolaxaphycin B (5) in DMSO (303K), Figure S3: DEPT135-NMR spectrum of [des-(Ala4-Hle5)] acyclolaxaphycin B (5) in DMSO (303K), Figure S4: TOCSY spectrum of [des-(Ala4-Hle5)] acyclolaxaphycin B (5) in DMSO (303K), Figure S5: ROESY spectrum of [des-(Ala4-Hle5)] acyclolaxaphycin B (5) in DMSO (303K), Figure S6: HSQC spectrum of [des-(Ala4-Hle5)] acyclolaxaphycin B (5) in DMSO (303K), Figure S7: HSQC-TOCSY spectrum of [des-(Ala4-Hle5)] acyclolaxaphycin B (5) in DMSO (303K), Figure S8: HMBC spectrum of [des-(Ala4-Hle5)] acyclolaxaphycin B (5) in DMSO (303K), Figure S9: 1H-NMR spectrum of [des-(Ala4-Hle5)] acyclolaxaphycin B3 (6) in DMSO (303K), FigureS10: 13C-NMR spectrum of [des-(Ala4-Hle5)] acyclolaxaphycin B3 (6) in DMSO (303K), Figure S11: DEPT135-NMR spectrum of [des-(Ala4-Hle5)] acyclolaxaphycin B3 (6) in DMSO (303K), Figure S12: TOCSY spectrum of [des-(Ala4-Hle5)] acyclolaxaphycin B3 (6) in DMSO (303K), Figure S13: ROESYspectrum of [des-(Ala4-Hle5)] acyclolaxaphycin B3 (6) in DMSO (303K), Figure S14: HSQCspectrum of [des-(Ala4-Hle5)] acyclolaxaphycin B3 (6) in DMSO (303K), Figure S15: HSQC-TOCSYspectrum of [des-(Ala4-Hle5)] acyclolaxaphycin B3 (6) in DMSO (303K), Figure S16: HMBCspectrum of [des-(Ala4-Hle5)] acyclolaxaphycin B3 (6) in DMSO (303K).
