# Supplementary material for: Biological Activities of Cyclic and Acyclic B-Type Laxaphycins in SH-SY5Y Human Neuroblastoma Cells
Source: Mar Drugs. 2020 Jul 15;18(7):364. doi: 10.3390/md18070364 (PMC7404270; doi:10.3390/md18070364)
Supplement: Supplementary file 1 [file marinedrugs-18-00364-s001.pdf]

Supplementary Materials:

Biological activities of cyclic and acyclic B-type laxaphycins in SH-SY5Y human neuroblastoma cells

Rebeca Alvariño[1], Eva Alonso[1,2 *], Louis Bornancin[3], Isabelle Bonnard[3,4], Nicolas Inguimbert[3,4], Bernard Banaigs[3,4] and Luis M Botana[1]

Figure S1. ^{1}H-NMR spectrum of [des-(Ala^{4}-Hle^{5})] acyclolaxaphycin B (**5**) in DMSO (303K) **Figure S2.** ^{13}C-NMR spectrum of [des-(Ala^{4}-Hle^{5})] acyclolaxaphycin B (**5**) in DMSO (303K) **Figure S3.** DEPT135-NMR spectrum of [des-(Ala^{4}-Hle^{5})] acyclolaxaphycin B (**5**) in DMSO (303K) **Figure S4.** TOCSY spectrum of [des-(Ala^{4}-Hle^{5})] acyclolaxaphycin B (**5**) in DMSO (303K)

Figure S5. ROESY spectrum of [des-(Ala^{4}-Hle^{5})] acyclolaxaphycin B (**5**) in DMSO (303K)

Figure S6. HSQC spectrum of [des-(Ala^{4}-Hle^{5})] acyclolaxaphycin B (**5**) in DMSO (303K)

Figure S7. HSQC-TOCSY spectrum of [des-(Ala^{4}-Hle^{5})] acyclolaxaphycin B (**5**) in DMSO (303K)

Figure S8. HMBC spectrum of [des-(Ala^{4}-Hle^{5})] acyclolaxaphycin B (**5**) in DMSO (303K)

Figure S9. ^{1}H-NMR spectrum of [des-(Ala^{4}-Hle^{5})] acyclolaxaphycin B3 (**6**) in DMSO (303K) **Figure S10.** ^{13}C-NMR spectrum of [des-(Ala^{4}-Hle^{5})] acyclolaxaphycin B3 (**6**) in DMSO (303K) **Figure S11.** DEPT135-NMR spectrum of [des-(Ala^{4}-Hle^{5})] acyclolaxaphycin B3 (**6**) in DMSO (303K) **Figure S12.** TOCSY spectrum of [des-(Ala^{4}-Hle^{5})] acyclolaxaphycin B3 (**6**) in DMSO (303K) **Figure S13.** ROESY spectrum of [des-(Ala^{4}-Hle^{5})] acyclolaxaphycin B3 (**6**) in DMSO (303K) **Figure S14.** HSQC spectrum of [des-(Ala^{4}-Hle^{5})] acyclolaxaphycin B3 (**6**) in DMSO (303K) **Figure S15.** HSQC-TOCSY spectrum of [des-(Ala^{4}-Hle^{5})] acyclolaxaphycin B3 (**6**) in DMSO (303K) **Figure S16.** HMBC spectrum of [des-(Ala^{4}-Hle^{5})] acyclolaxaphycin B3 (**6**) in DMSO (303K)

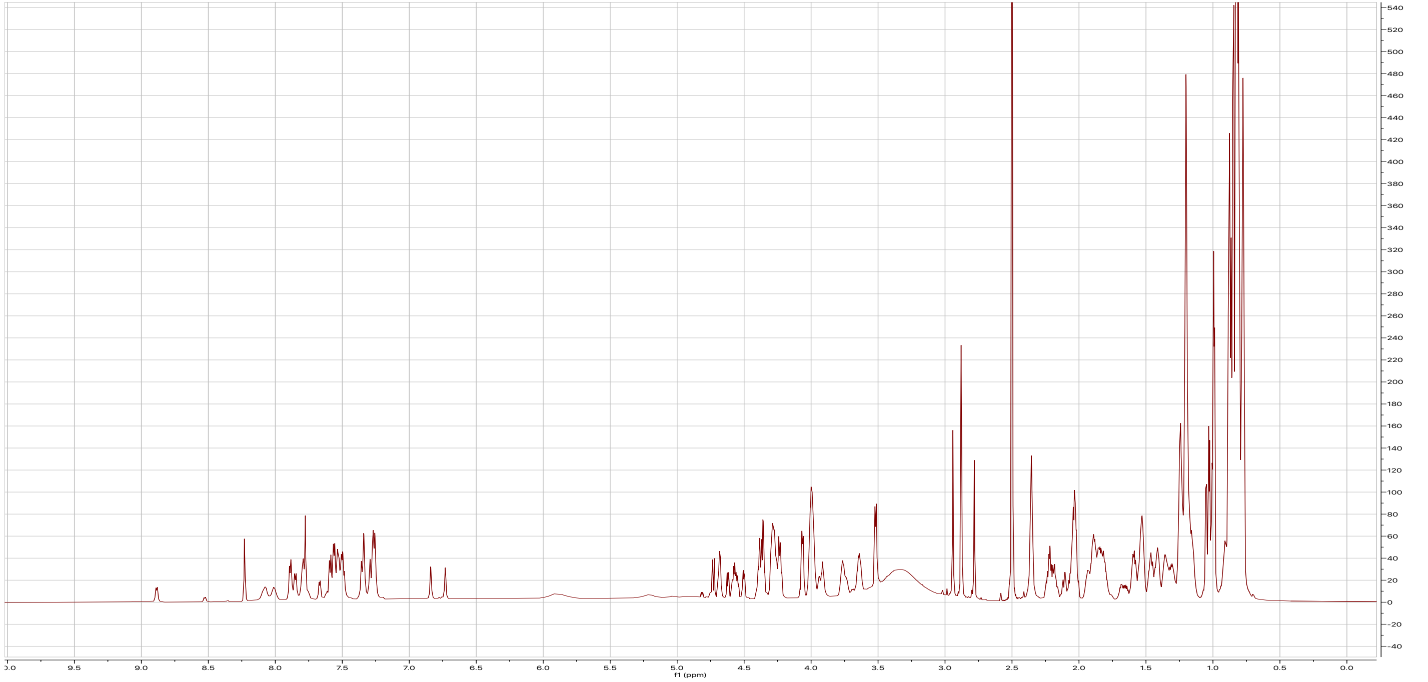

Figure S1. ^{1}H-NMR spectrum of [des-(Ala^{4}-Hle^{5})] acyclolaxaphycin B (**5**) in DMSO (303K)

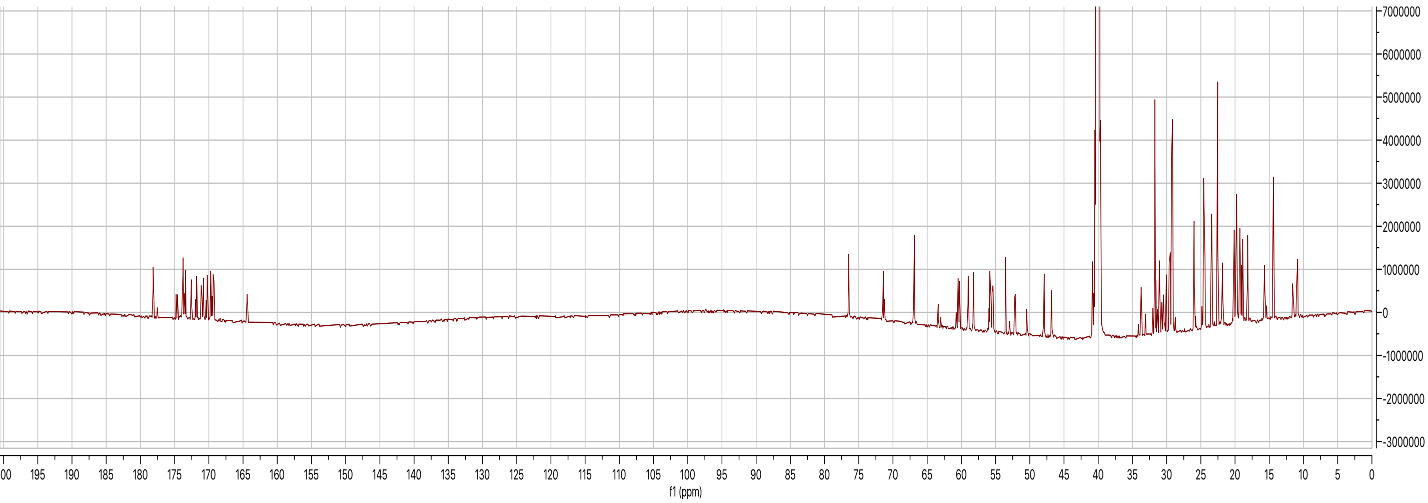

Figure S2. ^{13}C-NMR spectrum of [des-(Ala^{4}-Hle^{5})] acyclolaxaphycin B (**5**) in DMSO (303K)

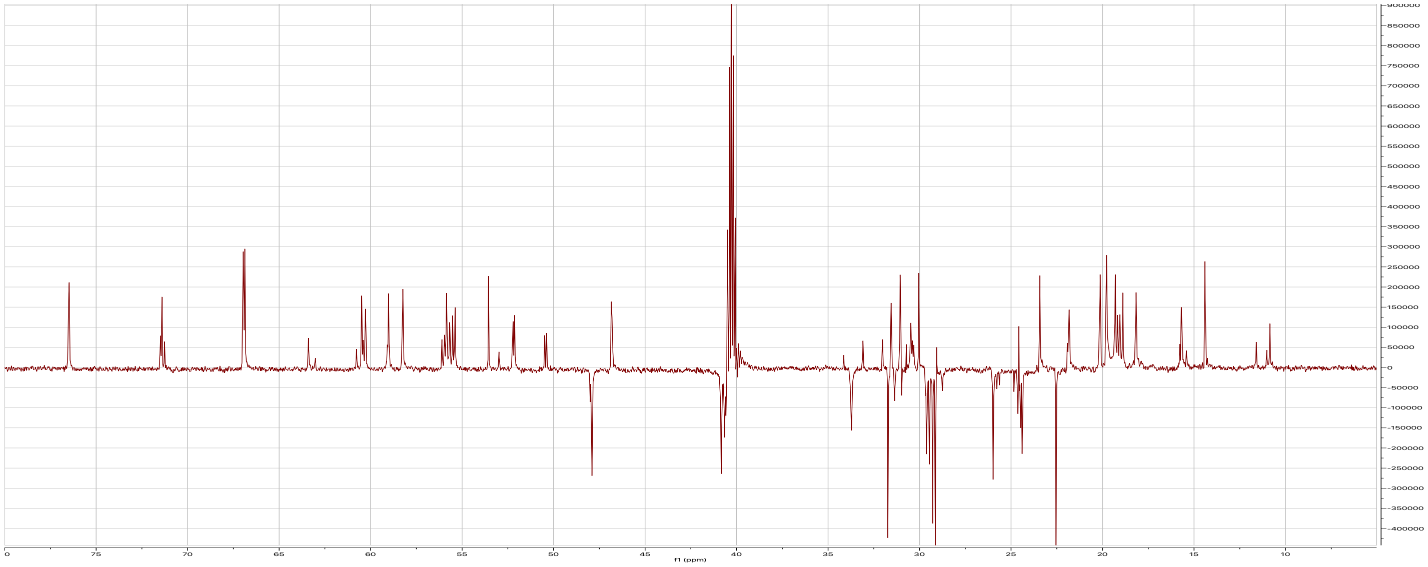

Figure S3. DEPT135-NMR spectrum of [des-(Ala^{4}-Hle^{5})] acyclolaxaphycin B (**5**) in DMSO (303K)

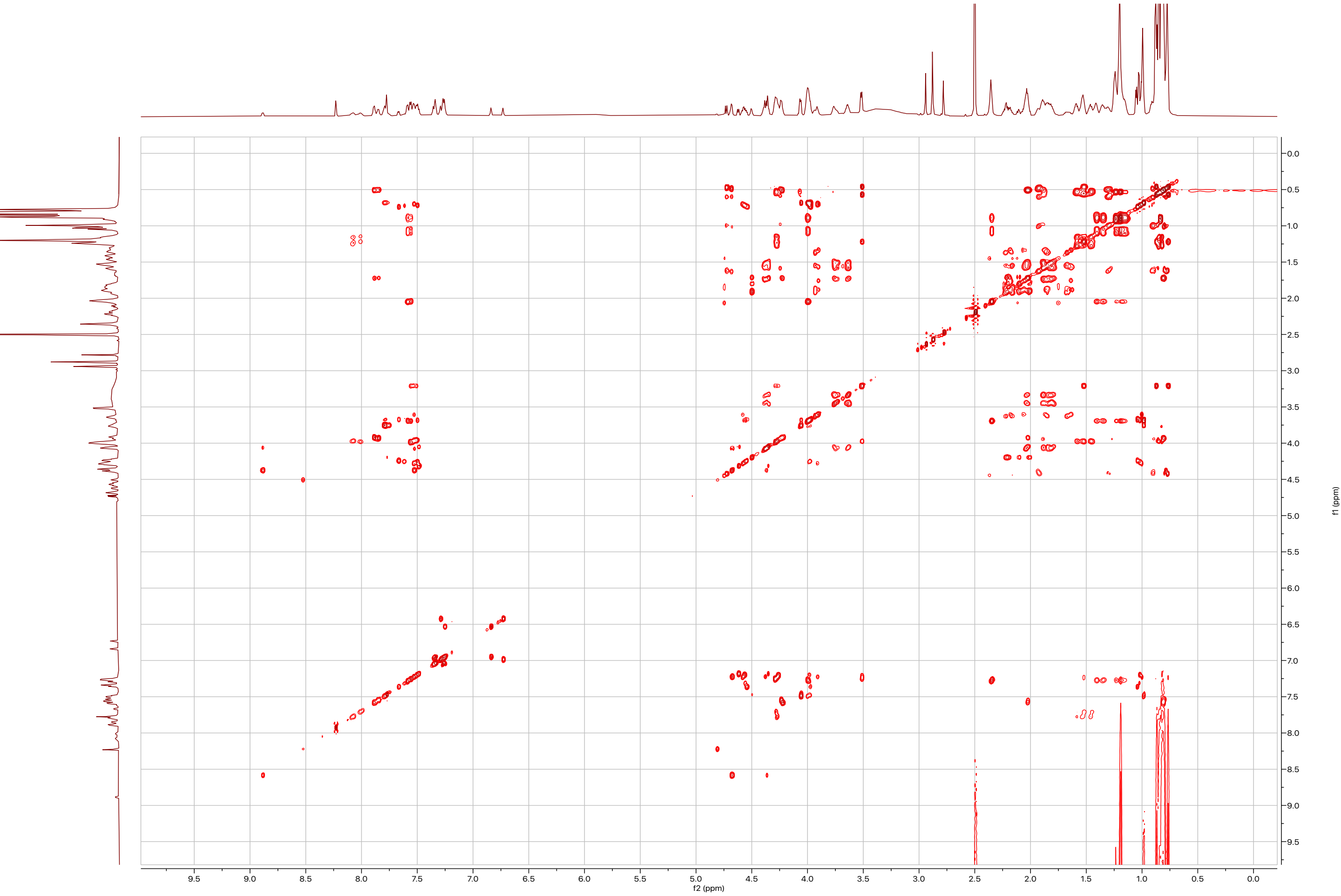

Figure S4. TOCSY spectrum of [des-(Ala^4-Hle^5)] acyclolaxaphycin B (**5**) in DMSO (303K)

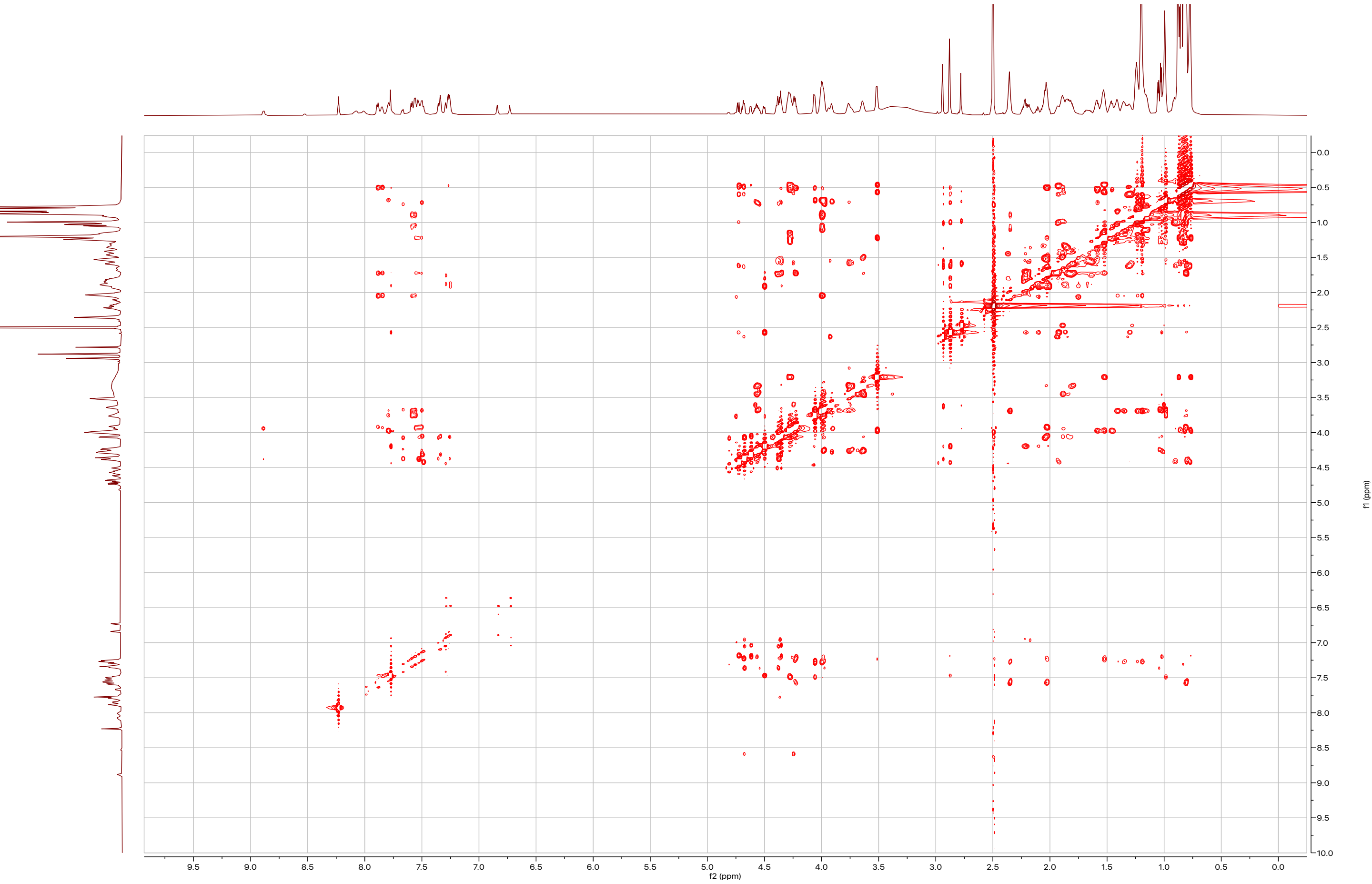

Figure S5. ROESY spectrum of [des-(Ala^4-Hle^5)] acyclolaxaphycin B (**5**) in DMSO (303K)

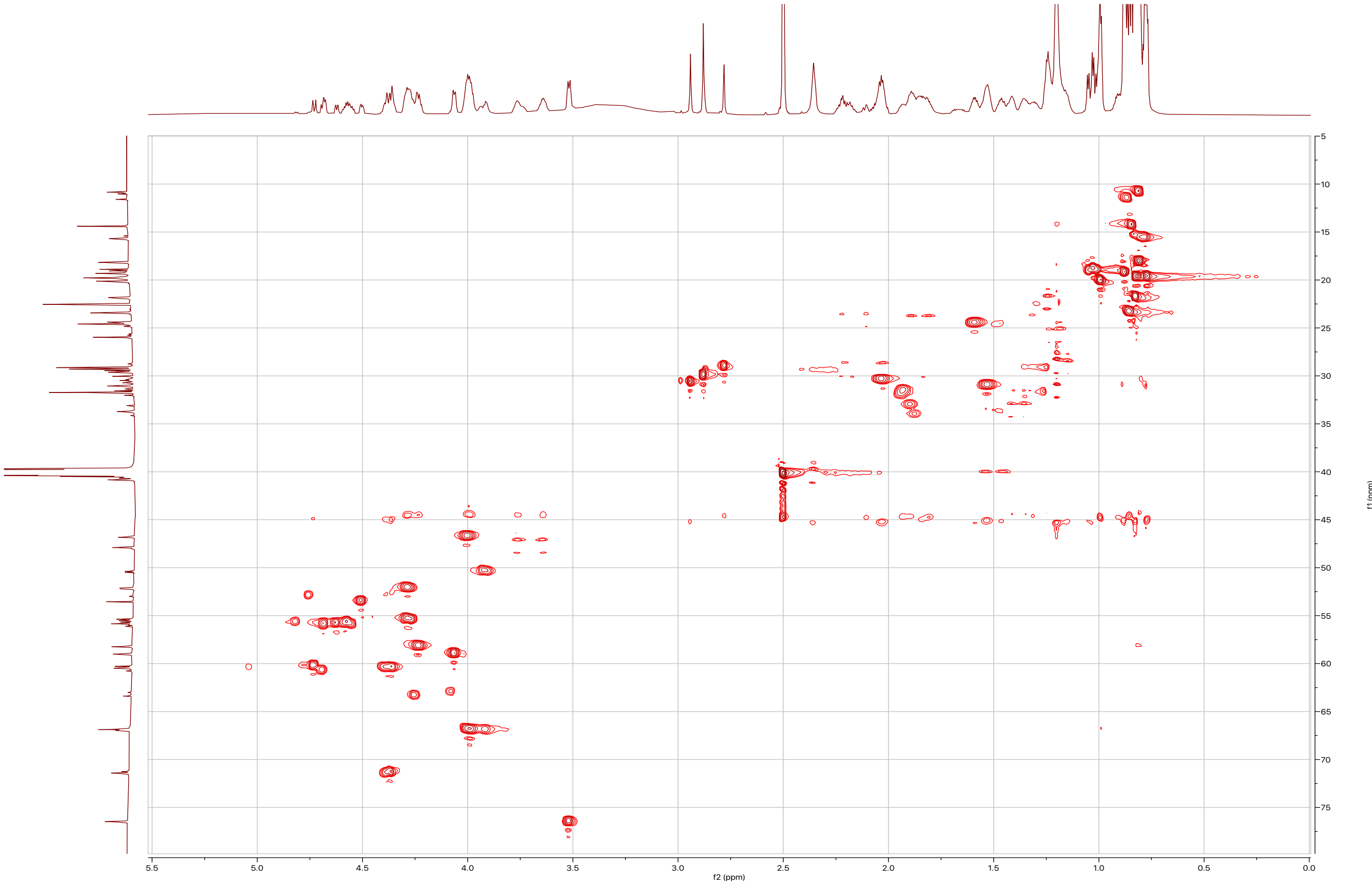

Figure S6. HSQC spectrum of [des-(Ala^{4}-Hle^{5})] acyclolaxaphycin B (**5**) in DMSO (303K)

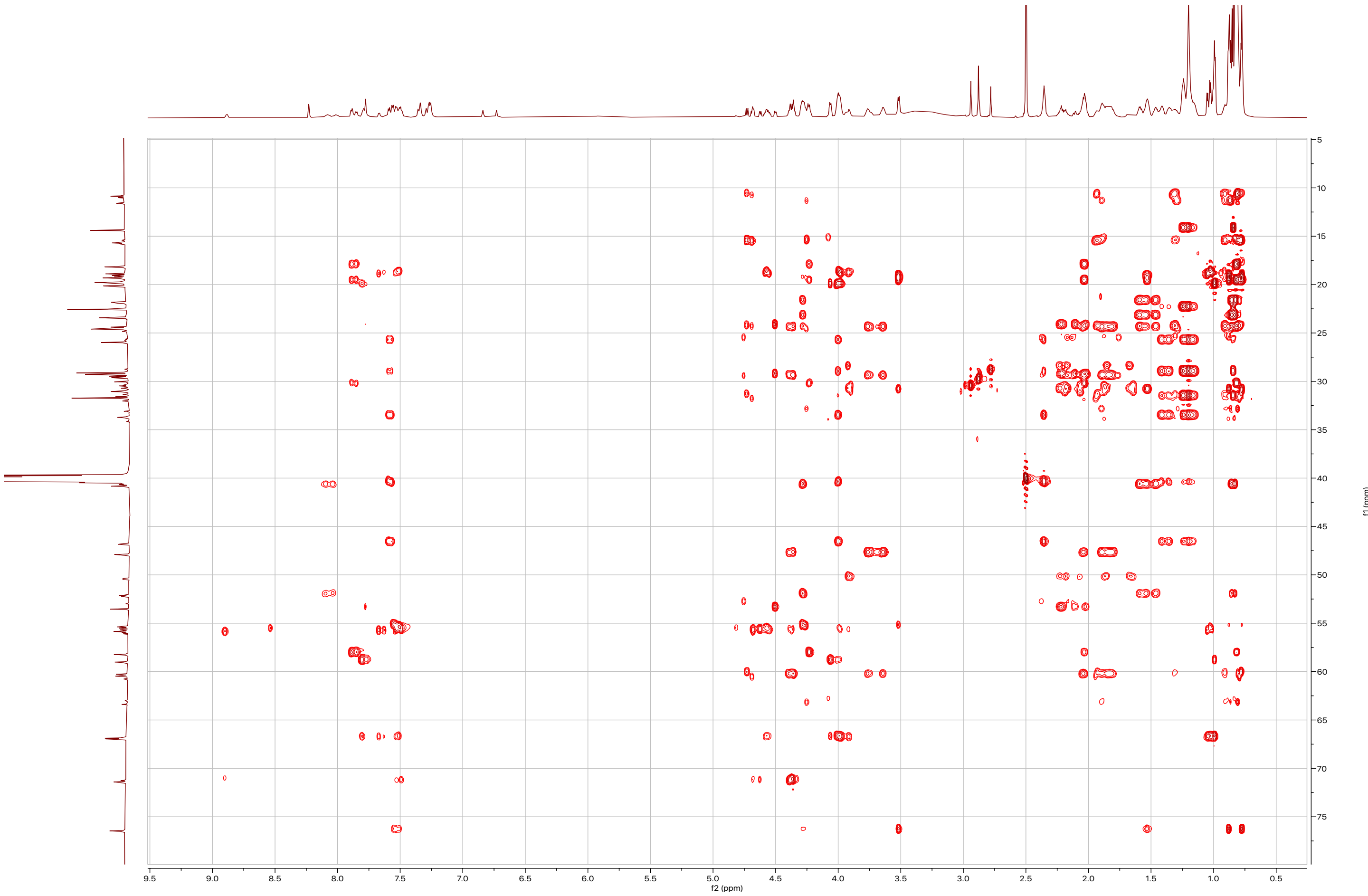

Figure S7. HSQC-TOCSY spectrum of [des-(Ala^{4}-Hle^{5})] acyclolaxaphycin B (**5**) in DMSO (303K)

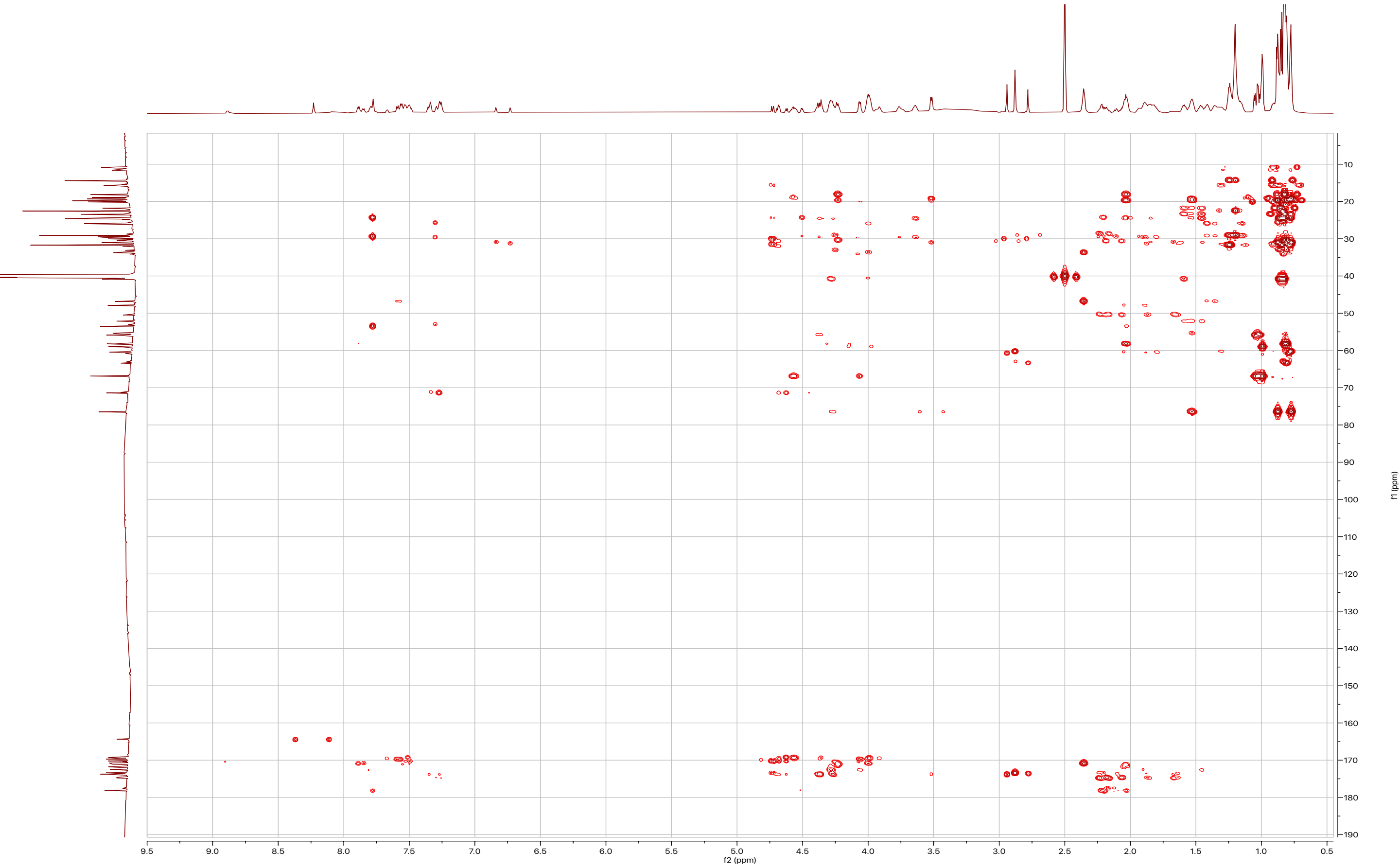

Figure S8. HMBC spectrum of [des-(Ala^4-Hle^5)] acyclolaxaphycin B (**5**) in DMSO (303K)

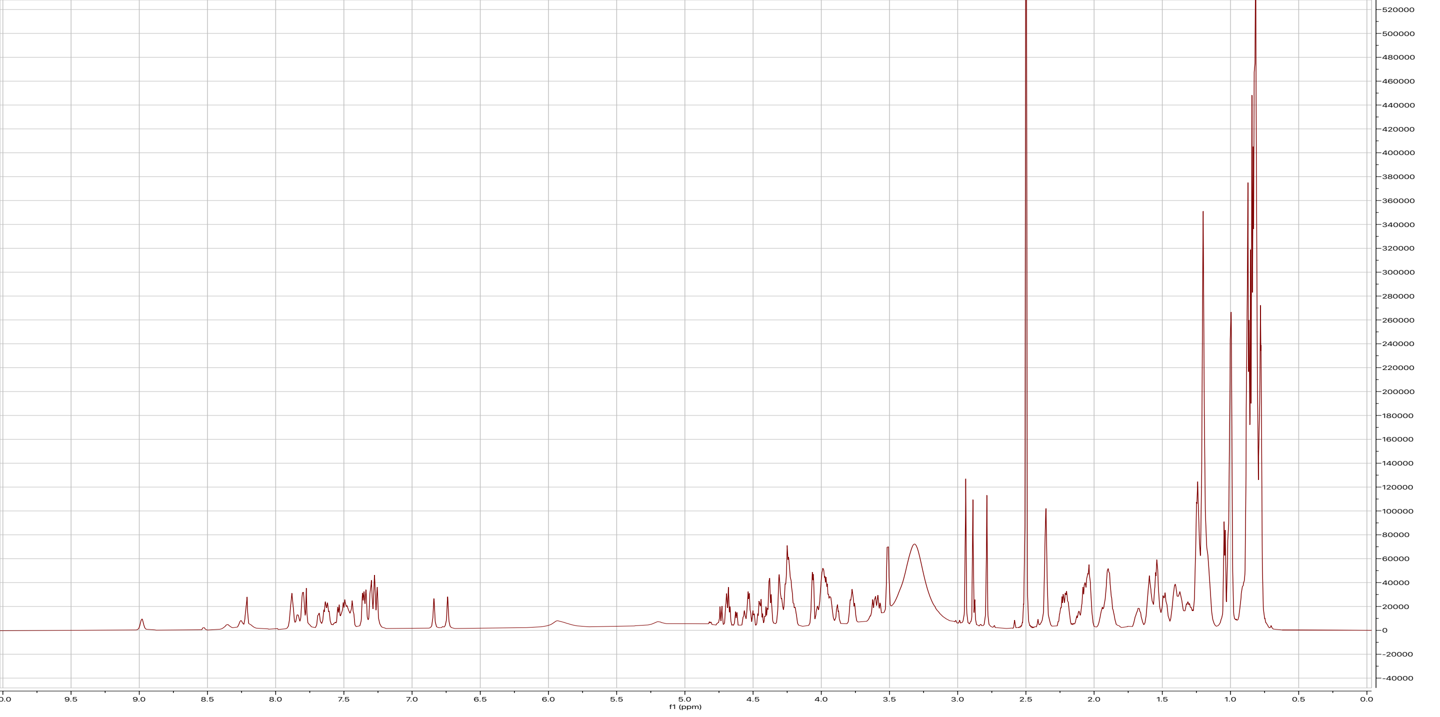

Figure S9. ^{1}H-NMR spectrum of [des-(Ala^{4}-Hle^{5})] acyclolaxaphycin B3 (**6**) in DMSO (303K)

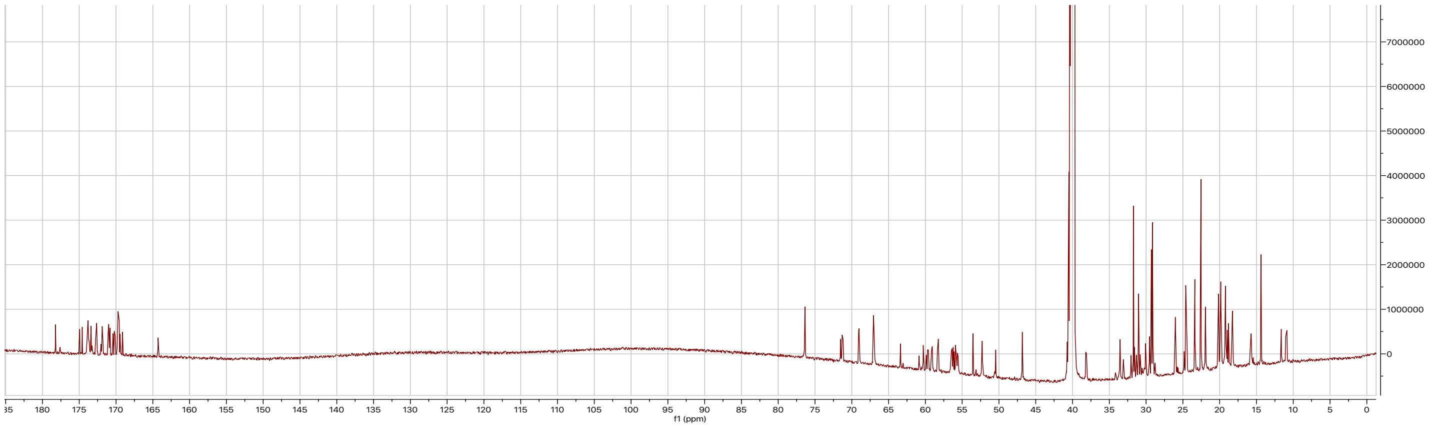

Figure S10. ^{13}C-NMR spectrum of [des-(Ala^{4}-Hle^{5})] acyclolaxaphycin B3 (**6**) in DMSO (303K)

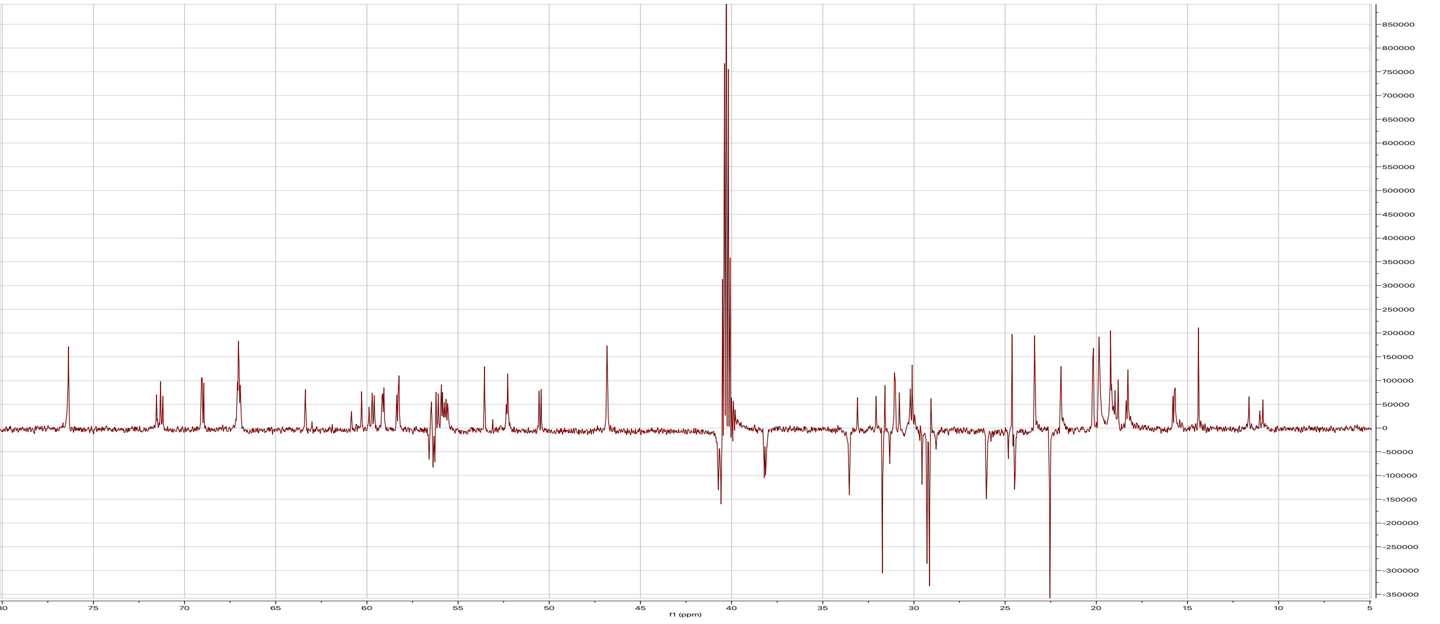

Figure S11. DEPT135-NMR spectrum of [des-(Ala^{4}-Hle^{5})] acyclolaxaphycin B3 (**6**) in DMSO (303K)

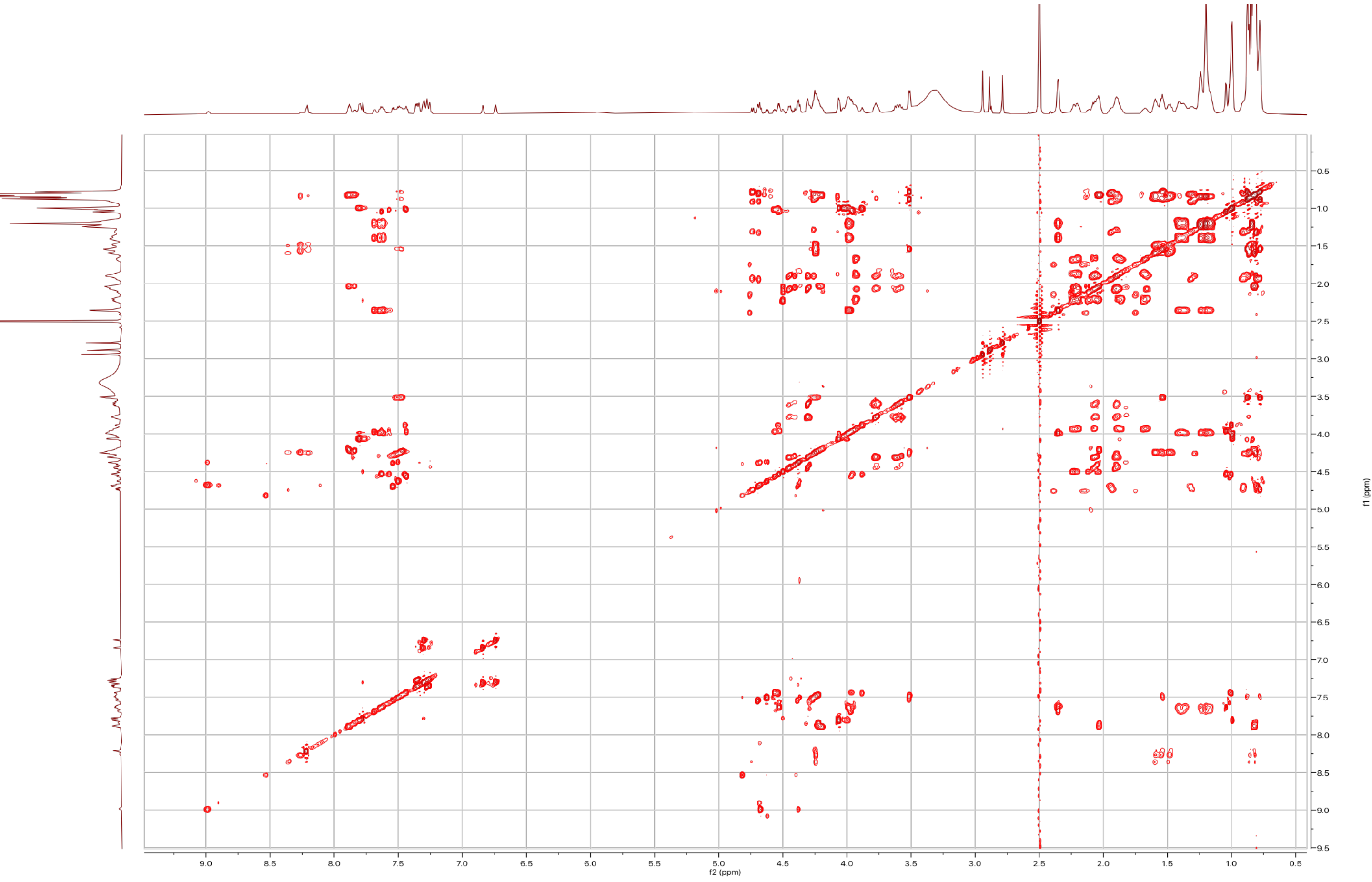

Figure S12. TOCSY spectrum of [des-(Ala^{4}-Hle^{5})] acyclolaxaphycin B3 (**6**) in DMSO (303K)

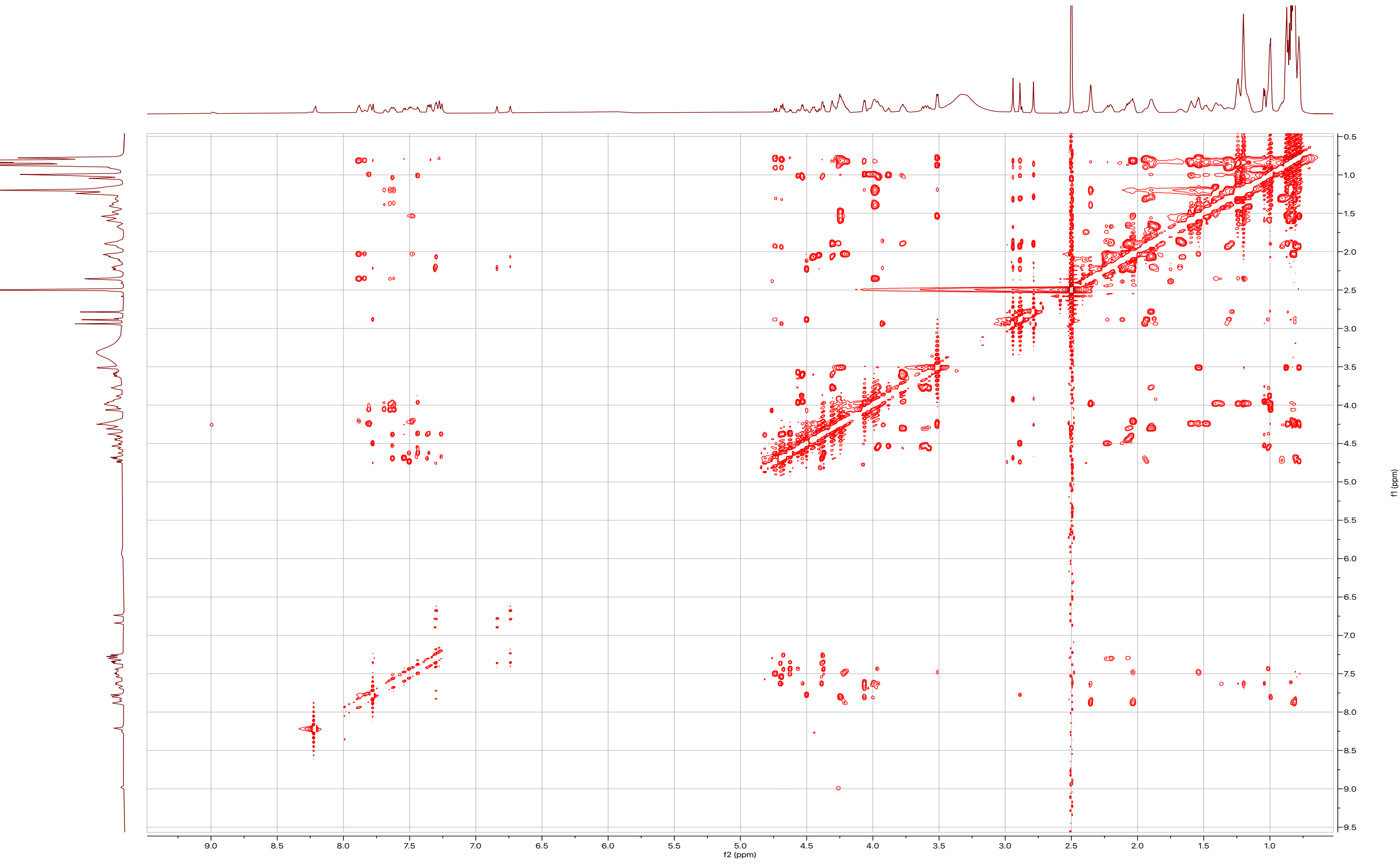

Figure S13. ROESY spectrum of [des-(Ala^{4}-Hle^{5})] acyclolaxaphycin B3 (**6**) in DMSO (303K)

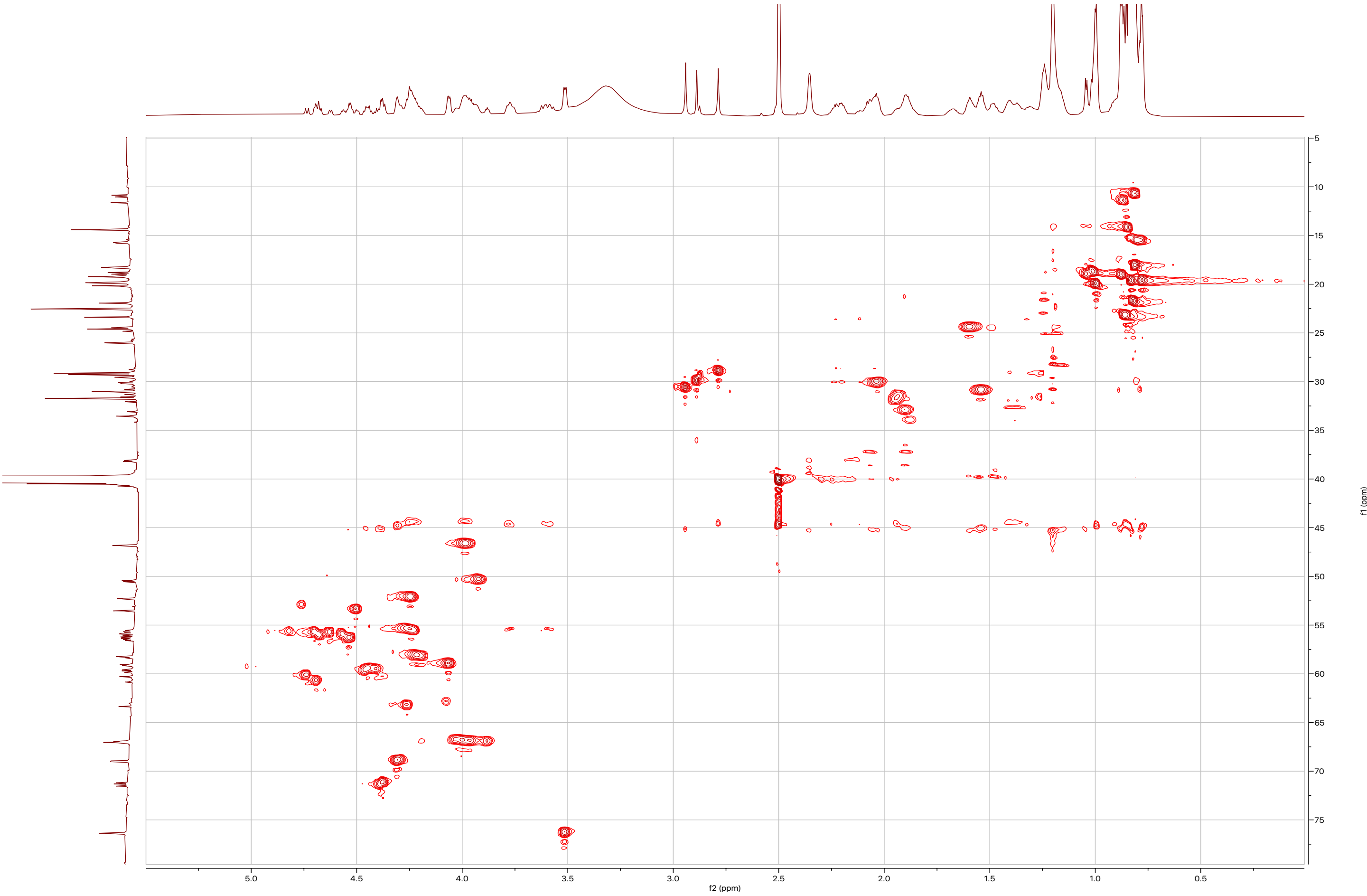

Figure S14. HSQC spectrum of [des-(Ala^4-Hle^5)] acyclolaxaphycin B3 (**6**) in DMSO (303K)

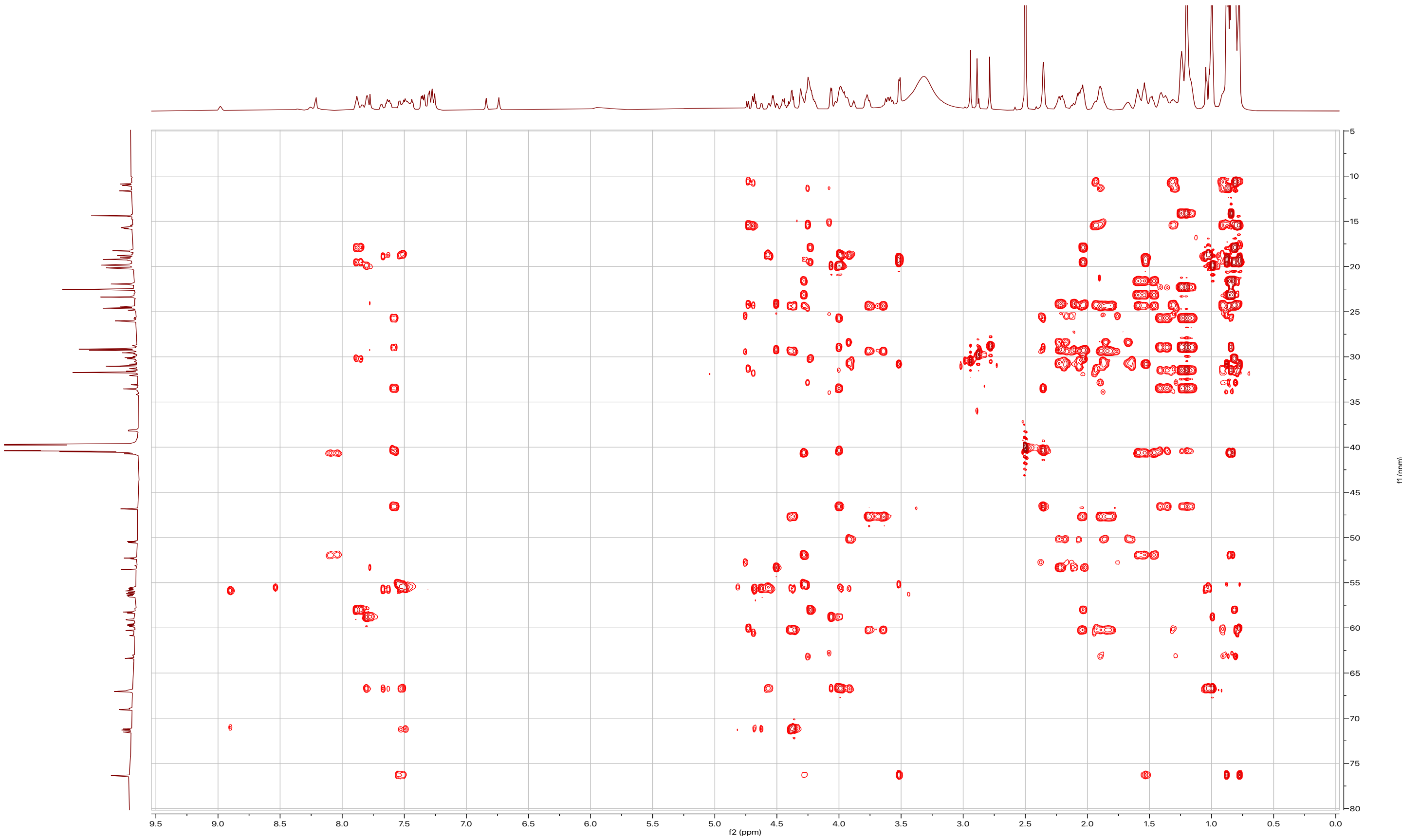

Figure S15. HSQC-TOCSY spectrum of [des-(Ala^4-Hle^5)] acyclolaxaphycin B3 (**6**) in DMSO (303K)

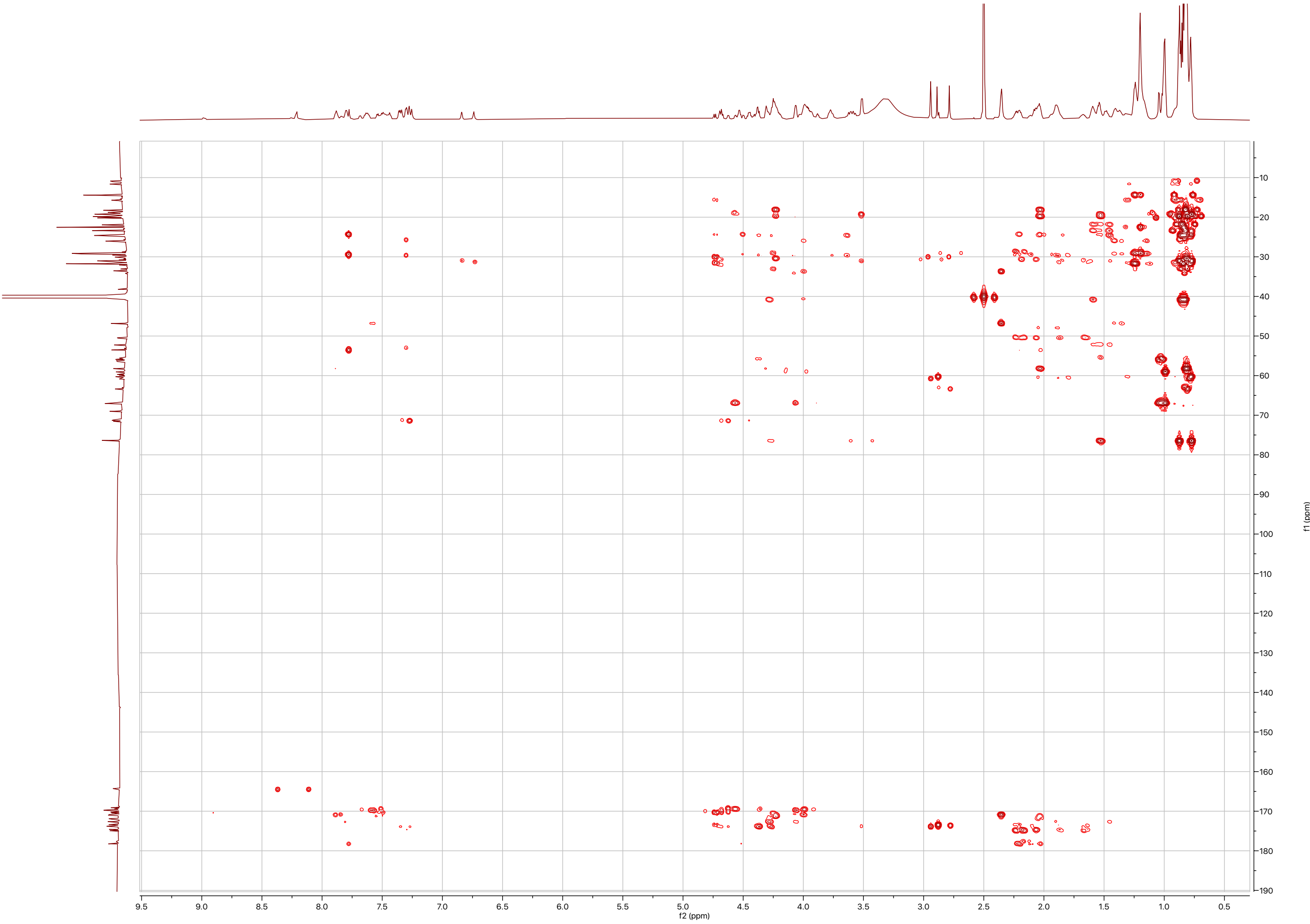

Figure S16. HMBC spectrum of [des-(Ala⁴-Hle⁵)] acyclolaxaphycin B3 (**6**) in DMSO (303K)
